# Renewed coexistence: learning from steering group stakeholders on a beaver reintroduction project in England

**DOI:** 10.1007/s10344-021-01555-6

**Published:** 2021-12-03

**Authors:** Roger E. Auster, Stewart W. Barr, Richard E. Brazier

**Affiliations:** College of Life and Environmental Sciences, University of Exeter, Streatham Campus, Amory Building, Rennes Drive, Exeter, EX4 4RJ UK

**Keywords:** Beaver, *Castor fiber*, Coexistence, Human dimensions, Reintroduction, Renewed coexistence, Stakeholder engagement

## Abstract

Species reintroductions are growing in popularity, and example motivations include supporting species populations or the restoration of ecosystem function. Interactions between humans and the reintroduced species are likely to occur post-reintroduction. Coexistence between humans and wildlife is adaptive and dynamic, in part requiring management of conflicts between humans and wildlife, or of conflicts between humans over wildlife management. We seek to learn from the experiences of steering group members in a Eurasian beaver (*Castor fiber*) project in England and identify how governance of coexistence with reintroduced species may differ from the governance of coexistence with species that are already present in the landscape. Using a qualitative thematic analysis of an online survey, we identify a series of lessons in six key areas: (1) project governance, (2) stakeholder engagement, (3) research and monitoring programme, (4) strategy to manage arising conflicts, (5) public engagement, and (6) broad perspectives on reintroduction trials. We advocate for reflective evaluation as an essential component of reintroduction projects to enable knowledge-sharing from experiences, leading to improved practices in the future. Reflecting on our analysis, we identify and define ‘Renewed Coexistence’—a new term that draws on pre-existing coexistence knowledge but identifies the unique elements that relate to governing coexistence with reintroduced species.

**Supplementary information:**

The online version contains supplementary material available at 10.1007/s10344-021-01555-6.

## Introduction

Coexistence between humans and wildlife ‘entails the behaviour of living together’ (Frank 2015). It is defined as adaptive and dynamic, but sustainable (Carter and Linnell 2016; König et al. 2020). Coexistence can be peaceful and beneficial, or it can be challenging; where interactions between humans and wildlife are more negative, human-wildlife conflicts can occur (Frank 2015; Nyhus 2016; Redpath et al. 2015). Conflicts may be real, or perceived by people (Bennett 2016; Messmer 2000). Management actions seek to prevent or mitigate conflicts and foster coexistence, but many human-wildlife conflicts are in truth human–human conflicts about wildlife or wildlife management (Marshall et al. 2007; Redpath et al. 2015).

Usually, coexistence refers to coexistence between humans and wildlife that is already present in the landscape. In wildlife reintroductions, however, there is a ‘new’ coexistence for the humans in the locality with a species with which they are unlikely to have prior experience. Wildlife reintroduction is the process of returning a species to an area where it was previously present but is now extinct (Seddon et al. 2007). It is a concept in conservation and ecological restoration that is growing in popularity (Corlett 2016). Motivations include boosting or supporting species populations, or facilitating restoration of ecosystem functioning (Seddon et al. 2014, 2015). For the latter, this is often associated with keystone species (which have disproportionately large effects on ecological community functioning (Hale and Koprowski 2018)) or ecosystem engineers (those creating or modifying habitats which affect both themselves and other organisms (Byers et al. 2006)).

The need to account for human dimensions in reintroduction is increasingly recognised. In many projects, there is potential for conflict between people and reintroduced species, or between people about reintroduction and management (Auster et al. 2020d; Hiroyasu et al. 2019; O’Rourke 2014). For example, although proposals for grey wolf (*Canis lupus*) reintroduction are favoured amongst the public in the western USA, some groups hold more negative views, e.g. farming and ranching groups whose economic interests may be affected by predation (Houston et al. 2010; Niemiec et al. 2020a; Sponarski et al. 2013; Williams et al. 2002). A study of attitudes towards wolf management in Colorado (where reintroduction is proposed) also found split opinions on acceptable management measures (Niemiec et al. 2020a).

If potential conflicts are not addressed, projects may fail (Auster et al. 2020b; Sutton 2015). E.g. Proposals to reintroduce Eurasian lynx (*Lynx lynx*) to England were rejected, partly as UK Government felt the efforts to engage with stakeholders and reduce concerns were insufficient (DEFRA 2018). The International Union for the Conservation of Nature (IUCN) has developed guidelines for reintroduction projects (IUCN and SSC 2013). For this reason, and alongside ecological considerations, they recommend understanding social factors: ‘…planning should accommodate the socioeconomic circumstances, community attitudes and values, motivations and expectations, behaviours and behavioural change, and the anticipated costs and benefits of the translocation’ (p11).

So far, there are limited examples of studies that relate to stakeholder engagement, conflict, and coexistence in the context of reintroductions. As they grow in popularity, there is a need to understand what may be similar or different in reintroduction, compared to fostering coexistence with species already present in the landscape. If a difference is identified, this knowledge would enable stakeholder perspectives to be better addressed, with coexistence between humans and the reintroduced species more likely to be fostered, if and where reintroductions take place.

In this study, we use an inductive thematic analysis of responses to a qualitative survey to record participant experiences of a reintroduction project. We aim to identify key factors that are informative for future reintroduction processes, discuss governance and stakeholder involvement in the context of reintroduction, and to identify what the implications of this may be for governing coexistence with reintroduced species compared to governing coexistence with a species that is already present in the landscape.

Structurally, the paper will first introduce Eurasian beavers, the focus of the reintroduction in question, before outlining the case study context. We will then describe methods and outline findings, demonstrating a series of lessons applicable for future reintroduction projects from the perspectives of practitioners, stakeholders and researchers involved. In our discussion, we will examine what these findings tell us about coexistence within the context of reintroductions. Finally, this will lead us to define a new term in response to our findings, which we hope will frame the thinking around coexistence and its application in reintroduction projects: renewed coexistence.

### Beavers in Great Britain

The Eurasian beaver (herein referred to as beavers) is a large mammal which lives in terrestrial aquatic environments and was historically present in Britain (Halley et al. 2020). They are often referred to as ecosystem engineers as they modify landscapes through dam-building and tree-felling behaviours (Campbell-Palmer et al. 2016; Stringer and Gaywood 2016). Beaver behaviours create habitats which support wider biodiversity (Law et al. 2019; Nummi et al. 2011, 2019; Nummi and Holopainen 2014, 2020; Stringer and Gaywood 2016; Ward and Prior 2020), and dams slow water flows through landscapes, reducing downstream flood risk and improving water quality (Brazier et al. 2020a, b; Brown et al. 2018; Graham et al. 2020; Puttock et al. 2017, 2018). Additionally, beaver tourism may benefit local businesses (Auster et al. 2020c; Campbell et al. 2007).

Conflicts with beavers are observed where they are present in continental Europe, such as water stored behind a dam upon agricultural land, or felled trees of social significance (Auster et al. 2020b; Campbell-Palmer et al. 2016). Mitigation techniques exist, e.g. dam removal, flow devices through dams, protective fencing, or compensation for damages (Campbell-Palmer et al. 2015, 2016; Morzillo and Needham 2015). There is also discussion about the relationship between beavers and fish, particularly salmonid migration (Auster et al. 2020a; Bylak and Kukuła 2018; Kemp et al. 2012; Malison and Halley 2020).

Beaver reintroduction is occurring in Great Britain at a nationally devolved level. Following a trial project in Argyll and monitoring of a population in Tayside, the Scottish Government legally protected beavers as a resident species (Coz and Young 2020; Gaywood 2018; Gaywood et al. 2015; Tayside Beaver Study Group 2015). In England, a population in Devon was monitored in a reintroduction trial (see below), and there are several fenced projects. In August 2020, UK Government announced the Devon beavers will remain, and a consultation recently closed on national approaches to reintroduction and management (UK Government 2020, 2021). In Wales, the ‘Welsh Beaver Project’ released beavers under licence into an enclosure in March 2021 (North Wales Wildlife Trust 2021).

### Study context: River Otter Beaver Trial

The River Otter Beaver Trial (ROBT) was a reintroduction trial in England between 2015 and 2020 in the catchment of the River Otter, Devon. The catchment is mostly rural with 50% of land use comprised of improved grassland, and 27% arable and horticulture. Only 5% is urban or suburban; human settlements are generally small and there are only three towns (Brazier et al. 2020a, p12).

Pre-2015, a small, free-living population of beavers was discovered in the catchment. The original population source was unknown. The beavers were to be removed but, following a locally driven campaign, Devon Wildlife Trust (DWT) was granted a 5-year licence to monitor the population (conditional on initial health-screening) (Crowley et al. 2017a; Natural England, 2015). The licence required evidence to be gathered on impacts across the 5 years, deemed a ‘trial’ phase. A monitoring plan was developed (Devon Wildlife Trust, 2017), with an exit strategy to terminate the project if triggers were met. The governance structure is comprised of several groups with defined roles (Table 1). Initially, organisations or individuals were invited to participate by Devon Wildlife Trust as the project leads, but others could be recommended or requested to join these groups.

**Table 1:** Summary of the ROBT project governance structure

Hierarchy level	Group	Role	Members/participants	Chair
1	**Licence group**	To monitor compliance with the licence	Statutory agencies, local authorities, trial partners	Natural England
2	**Project management group**	Responsible for day-to-day delivery and management of the Trial	Partner organisations	DWT
2	**Steering group**	To provide oversight from key stakeholders and provide Project Management Group with scrutiny, advice, and support. Key role to assess exit strategy triggers annually	High-level representation from wide range of key stakeholder groups	DWT
3	**Beaver management strategy framework working group**	Formed by steering group and tasked with development of post-2020 beaver management strategy framework	Subset of SG members	DWT
3	**Science and evidence forum**	Oversee development and delivery of monitoring plan, in an objective and scientific manner To publish *Science and Evidence Report* summarising research findings	Academic researchers and other stakeholders involved in monitoring and evidence gathering	University of Exeter
3	**Fisheries advisory forum**	Specialist group to advise ROBT in respect to fisheries interests	Key national and local fisheries organisations and syndicates	Clinton Devon Estates
3	**Community and education forum**	Public information exchange	Local community members, ROBT volunteers, landowners within trial catchment	Devon County Councillor
*3*	*Internal DWT Communications Group*	*Coordinate communications and fundraising*	*DWT*	

In 2020, findings were presented in the *Science and Evidence Report* (Brazier et al. 2020a)*.* A proposed *Post-2020 Beaver Management Strategy Framework* was also developed (River Otter Beaver Trial 2019) in case the beavers could remain. These were presented to UK Government in February 2020. The following August, the beavers were permitted to remain permanently and disperse naturally (UK Government 2020).

The ROBT was funded by donations and fundraising led by DWT and did not receive government funding. (Details are available in Brazier et al. 2020a (back cover)).

Throughout the ROBT, social research efforts engaged with community stakeholders, including:

- a broad nationwide survey of public attitudes to beaver reintroduction (Auster et al. 2020d).
- focused study with individuals who reported beaver conflicts with land/property (Auster et al. 2020b).
- investigation into beaver tourism and its reception amongst residents and businesses (Auster et al. 2020c).
- exploration of perspectives of beaver reintroduction among anglers in the River Otter catchment (Auster et al. 2020a).

## Methods

We conducted an online survey of stakeholders involved in ROBT governance. Participants were questioned on a range of areas relating to the trial, and a qualitative inductive thematic analysis enabled us to recognise key features in the responses. This method allowed us to conduct research in a safe, remote manner during the COVID-19 pandemic and national lockdown restrictions; participants may have had varying priorities, and this enabled participation in their own time, from home.

### Survey design

We proposed the study to a Steering Group (SG) meeting on 13th February 2020 (prior to COVID-19 restrictions), at which SG members supported the proposal. Members indicated a preference for a questionnaire-based study to facilitate participation around work commitments. Proposed topic coverage was determined pragmatically and outlined to the SG in the meeting as a set of ‘key questions’ in the proposal presentation (Supplementary Information). The proposals were approved, with additional comment that the questionnaire should also ask about risks involved in steering group participation.

We designed the survey in Qualtrics software. Questions were based around the key questions presented to the SG and their response. We piloted the survey internally to ensure balanced question framing and coverage. We anticipated participation would take up to 30 min. Questions (Supplementary Information) were designed to ask respondents about: their ROBT involvement, including risk and challenges, perspectives on trial governance, views on trial successes or failures, whether participation was perceived as of value, lessons for the future, and whether they would consider participating in future projects.

### Participants and survey distribution

Originally, this was proposed for steering group (SG) members only as key informants. This group comprised of a wide range of stakeholders with high levels of representation and knowledge of their organisational interests who had not had opportunity to participate in the earlier social studies (outlined in Study Context). At the 13th February 2020 meeting, SG members requested the invitation be extended to the Beaver Management Strategy Framework Working Group (BMSF) and Science and Evidence Forum (SandE), thus including groups responsible for research, monitoring, and developing key document outputs (Brazier et al. 2020a; River Otter Beaver Trial 2019). Hence, at the SG’s request, we circulated the invitation to participating members of the three groups responsible for steering the ROBT and document outputs (SG, BMSF, and SandE).

We sent the invitation on 30th April 2020, followed by two reminders. The survey closed on 10th August 2020. This included an extended deadline as we recognised possible impacts of the COVID-19 pandemic upon participants.

**Fig. 1:**
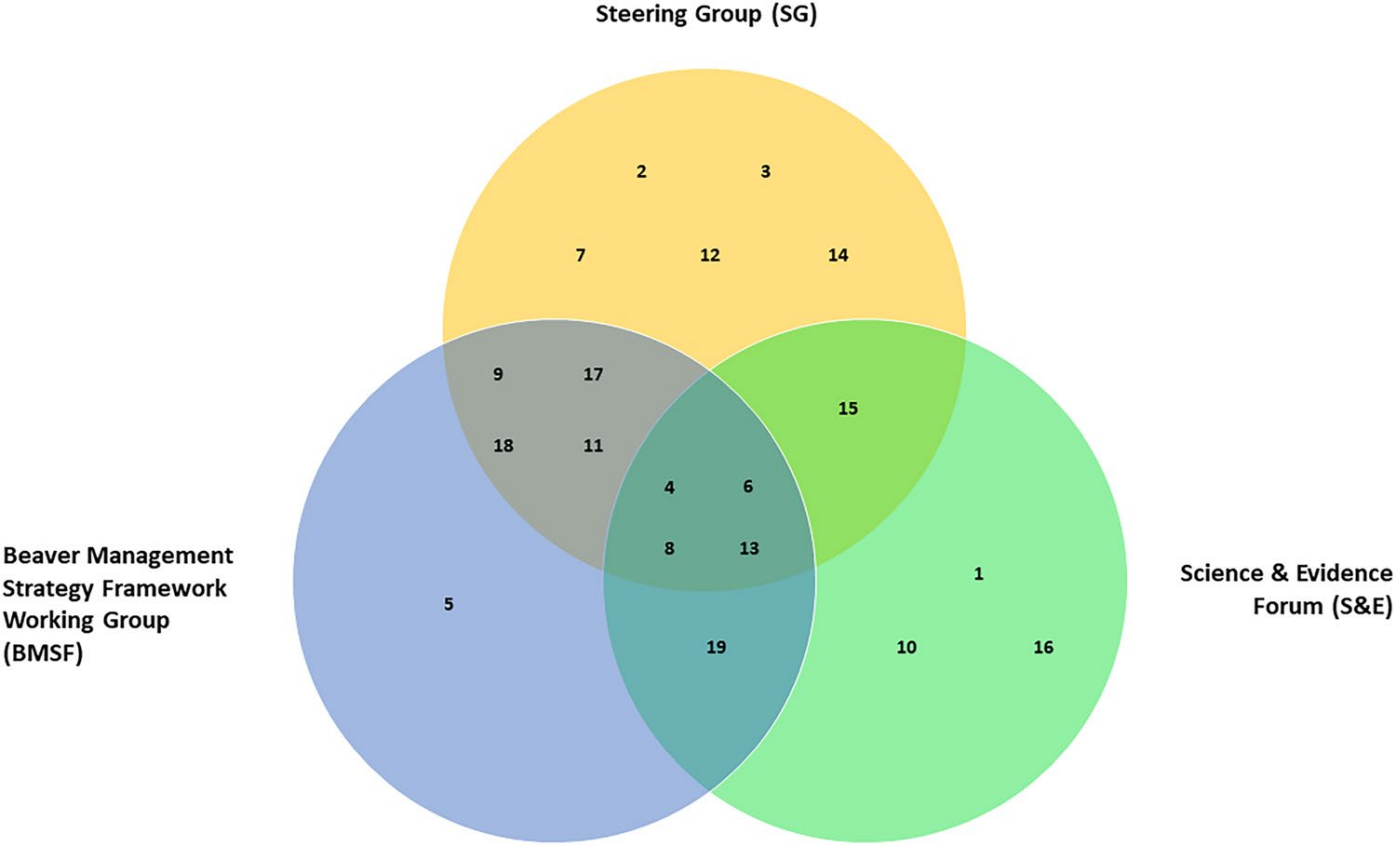
Illustration of the groups upon which participants sat, using assigned participant numbers

For data protection purposes, the invitation was email circulated on our behalf by DWT who had access to members’ contact details. We were informed of a potential pool of 26 respondents. We received 19 responses (73%): fourteen SG, ten BMSF, and nine SandE members (some participants sat in multiple groups; see Fig. 1). Further participant details are given in Appendix 1.

Following the invitation, we received a participation request from an individual who had regularly engaged with the ROBT. Considering their trial involvement, we accepted. However, the individual was not a member of the focal groups and, as other people outside of these groups did not have the same opportunity, their responses were not included in the primary analysis. Their responses are however made available in the data (Supplementary Information), identified as Participant 20.

Whilst this is a small number of participants, which could limit the empirical generalisability of our findings, the participants are key informants in the case study setting and represent members of the project's Steering Group, which itself is argued to be cross-sectoral and thus representative of a wide range of stakeholders. Our qualitative method allowed us to provide an understanding of the processes in the situation and perspectives of those involved (Firestone 1993; Tsang 2013). We relate our results to existing literature and believe they provide a deep understanding that is informative for further reintroduction contexts.

### Researcher positionality

Researcher positionality was an important consideration; the lead author (who also led analysis) had been an SandE member and conducted previous research within the ROBT (detailed in the papers listed in the introduction, and Brazier et al. 2020a), so it was a possibility that the lead author’s experiences, or views could have influenced study findings. Several factors were employed to minimise this potential and ensure objectivity:

- the study was developed in discussion with the first co-author (an academic, independent from the ROBT) and piloted with two colleagues who had no ROBT involvement;
- the lead author was excluded from participation;
- a ‘Findings Report’ of key points (Supplementary Information) was shared with participants to comment between 27th November and 21st December 2020;
- anonymised participant responses are available in full in the Supplementary Information;
- the final text was subjected to peer review.

Furthermore, the thematic analysis used an inductive approach to coding data (see Sect. 2.5). Although a researcher will always play an active role in reporting findings, this data-driven coding process meant resulting themes are strongly linked to the data, rather than driven by the researchers’ theoretical interests or analytical preconceptions (Braun and Clarke 2006).

Additionally, the second co-author sat in the groups so was excluded completely from survey design and had no input on analysis or findings (beyond opportunity provided for all participants to comment on the Findings Report). They contributed by checking study context details and reviewing structure and presentation of material. The funders had no study oversight.

### Ethics

Prior to taking part, respondents were given details of the study and informed that participation would be voluntary and anonymous (the full information provided to participants is available in Supplementary Information). All participants gave written consent by ticking a box to indicate that they had read and agreed to this information to participate; this box was a required field to proceed with the survey. In recognition of COVID-19 pandemic circumstances, we emphasised the voluntary nature of participation.

### Analysis

We used qualitative thematic analysis to identify key themes in the data, following the process described by Castleberry and Nolen (2018). This involved first coding survey transcripts—the disassembly of raw data into usable data (codes) by identifying features within the text. Codes were generated from the data. The text could be coded under multiple codes. We had an initial long list of 272 codes.

**Fig. 2:**
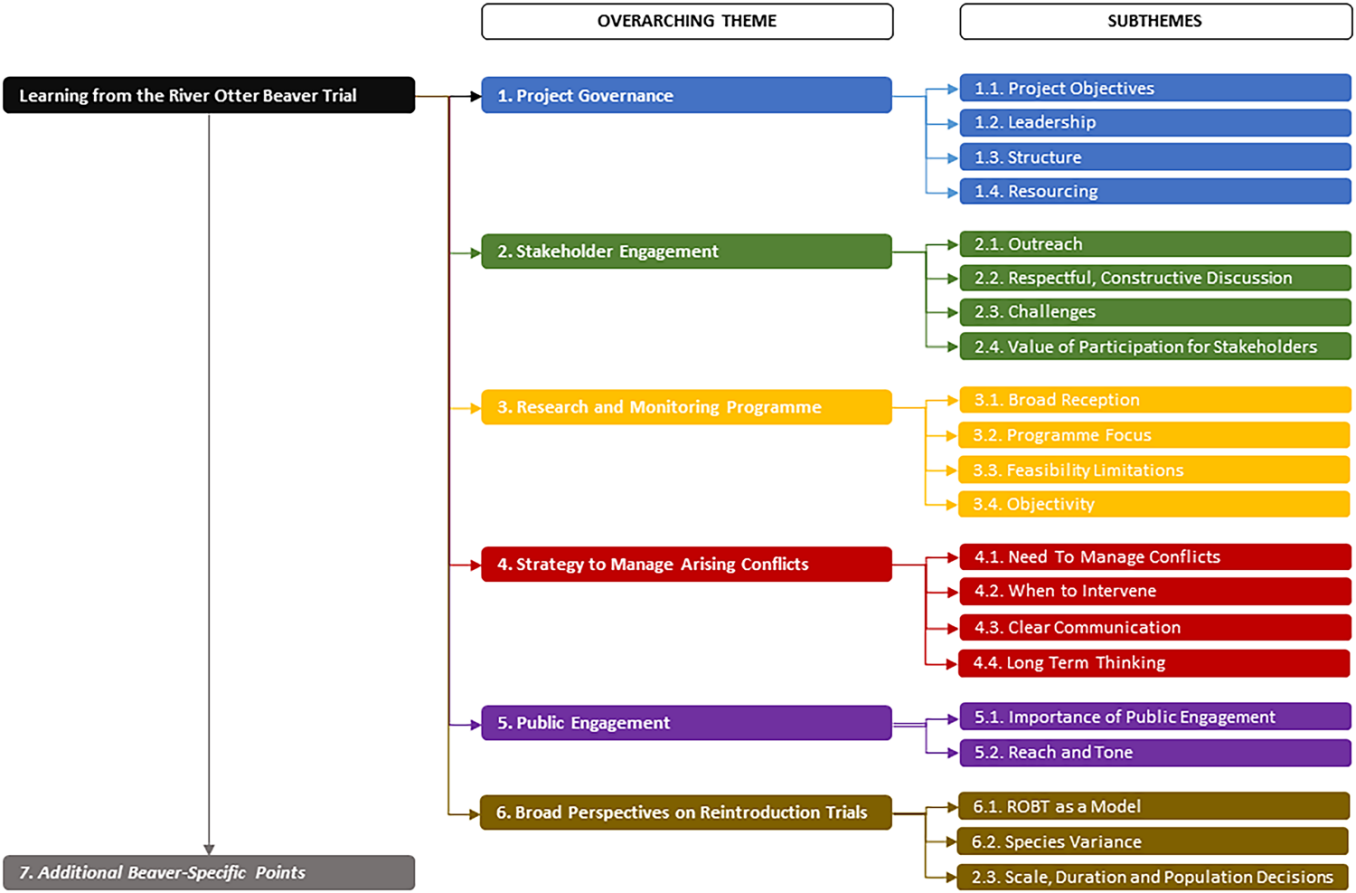
Summary of over-arching themes formed of their respective subthemes

We reviewed the long list and identified similarities and differences to re-assemble codes—rearranging them into context with one another. This generated 22 preliminary themes. We subjected these to a second round of re-assembly to generate six overarching themes. Under these, 21 of the preliminary themes formed subthemes (Fig. 2). The remaining preliminary theme (additional beaver-specific points) consists of extra points unique to beavers, so is summarised in Appendix 2.

## Identified themes

We identified six overarching themes identifying lessons for future reintroduction projects: (1) project governance, (2) stakeholder engagement, (3) research and monitoring programme, (4) strategy to manage arising conflicts, (5) public engagement, and (6) broad perspectives on reintroduction trials. Each is outlined in this section, using the participants’ words and relevant literature.

We refer to participants using their participant numbers, i.e. P1 = participant 1, P2 = participant 2.

### Project governance

First, participants identified a need for clearly defined objectives. Clear objectives facilitate successful planning and assessment (Ewen et al. 2014) but must recognise what is feasible within the project scope (see theme 6), with expectations managed accordingly. This may require defined timescales to ‘measure if the objectives are met’ (P8). P16 said future reintroductions need to consider ‘The need for realistic expectations of what can be achieved during a relatively short trial reintroduction (perhaps particularly the expectations of various stakeholders)’. In the ROBT, P13 felt the focus of the project was upon a holistic understanding of beaver reintroduction, whilst P11 felt the focus to be on conservation, whilst not factoring in other land management changes locally.

Second, reintroduction projects require committed leadership, a point also recognised in a study of white-tailed sea eagle reintroduction in Ireland (Sutton 2015). P4 stated ‘running a successful project requires huge focus and dedication far and above the normal 9–5 working practices’, and P2 cited ‘The very strong lead provided by DWT and their unstinting commitment to the project’ as a success. Leadership will need to take an honest and transparent approach, recognising both benefits and conflicts. This can be important for building and maintaining trust between project leads and stakeholders (Auster et al. 2020b; Madden, 2004; Riley et al. 2018). For example, P4 indicated ‘being open and honest about the conflicts that beavers can cause has helped with lots of stakeholder groups’. However, P17 believed that ‘possible negative impacts on fish were somewhat glossed over’ which they cited as an ‘example to warn against brushing issues like this aside, in terms of maintaining stakeholder engagement’*.* One consideration put forward by participants towards an objective approach may be for independent chairing of governance groups; P9 and P11 believed the ‘various groups should have had independent chairs’ (P11). If this is not possible, the transparency of leadership will increase in importance; there may always be factors which affect trust in wildlife managers but where stakeholders perceive greater transparency, trust levels are likely to increase (Riley et al. 2018). If concerns are not addressed and only extreme positive messaging is utilised, there is a risk of increased conflict and opposition (Niemiec et al. 2020b).

Third, the wider trial structure (Table 1) received favourable comments. For example, P10 said ‘I believe the trial structure was appropriate and communication between groups was managed very well. There was enough cross-over in terms of the same people sitting on different groups to ensure that concerns/questions were raised across all relevant groups’. However, some felt the structure was complicated, with P3 reporting that the framework could be simplified, and P2 felt there was some duplication of effort. Hence, we suggest that reintroduction project governance frameworks (or group responsibilities) should be clearly defined to ensure aims can be met. This may include defining direct relationships between organisations; in one case here, a formal memorandum of understanding was agreed between the ROBT and P8’s national organisation, which P8 found ‘very helpful in defining roles and responsibilities’.

Fourth, defining responsibilities may reduce duplication of effort, increasing efficiency. This is important as ‘Resource demands and commitments’ (P7) were highlighted as a challenge in governing the project, requiring ‘personnel, time and money’ (P16). Any similar reintroduction should consider efficient resource use, with P18 stating the Trial could be ‘streamlined’ (see theme 6). Costs of reintroduction can be high (Hilbers et al. 2019). This may include financial risks for project leads. In the ROBT, DWT held responsibility for costs of negative beaver impacts under licence conditions issued by Natural England. ‘There have also been financial risks associated with the Trial—in particular the resources that had to be put aside for implementing the Exit Strategy, and for compensating for any significant impacts—which may need to have been covered by our insurance (e.g. Flooding of properties that [DWT] could have been responsible for under the licence)’ (P4). However, P3 believed costs should be viewed as a future investment, rather than purely as a costly process: ‘[this] probably has to be seen in terms of recovery of beavers across UK […] and not as a cost simply to recovery [on the] River Otter, or Devon and South west’.

### Stakeholder engagement

Identifying key stakeholders and understanding their perspectives is vital for reducing conflicts and fostering coexistence (Coz and Young 2020; König et al. 2020; Redpath et al. 2013). Where failure to account occurs, conflicts could arise in reintroductions also (Auster et al. 2020d; IUCN and SSC, 2013; O’Rourke 2014). In the ROBT, stakeholder engagement was seen by most as strong. When asked about Trial successes, thirteen participants cited stakeholder engagement or an element of it. For example, P2 said there were ‘good opportunities for active participation/input by relevant stakeholders’, and P8 said meetings were well run, ‘inviting full participation […] on every occasion’. Seven participants referred to invited stakeholders as being of a broad range of interests, which was received positively. P7 reported this to include landowners, business interests, environmental groups, and members with a social or community interest, which they then said felt ‘well balanced’. Thus, there was opportunity for key stakeholder representation. We suggest future reintroduction projects make similar concerted efforts to engage with the breadth of identified stakeholders (Auster et al. 2020d; Coz and Young 2020).

Along with identifying stakeholders, engagement methods should be considered. It has been shown in the human-wildlife conflict literature that, where stakeholders feel their views or concerns are being taken seriously, trust between parties can be fostered. This enables issues to be shared collectively, with conflicts addressed or prevented early (Auster et al. 2020b; Decker et al. 2015, 2016; Redpath et al. 2015, 2013; Riley et al. 2018). In the ROBT, P19 cited successes in ‘Listening, treating all concerns seriously, trust and good communication’. P13 referred to ‘Excellent partnership working across a wide range of stakeholders who did not necessarily agree about the reintroduction of beavers’. Not all partners shared the same viewpoint, but it was reported they worked together to find solutions: ‘one important point is that the successful operation of the Steering Group was the willingness of the individual participants to engage positively (i.e. highlighting relevant concerns as necessary, but in a manner which sought to resolve these in a mutually acceptable manner). There were tensions between various stakeholders involved in or affected by the Trial, but the Steering Group helped to manage these well’ (P2). Further, respectful discussion can enable different parties to learn from each other. For example, P3 stated there was ‘Dissemination of much deeper understanding throughout group…from beaver ecology to farmers and fishermen for example, but also the reverse, from fishing concerns and preoccupations and local economic interests and constraints upwards to beaver enthusiasts and ecologists. All such groups benefit from this flow and counterflow of understanding’.

However, challenges can be encountered in stakeholder engagement. Here, four were reported and are described in Table 2. Despite challenges, participation in the ROBT was reported to be of value for those involved; we asked whether group members felt participation was of value for their respective organisations and all participants ticked ‘yes’. Reasoning included ‘strengthened stakeholder relations’ (P12), ‘participation in discussions’ (P9), being ‘better placed to field questions from local farmers’ (P5), ‘bolstered […] membership (potential future financial support) and reputation for completing high-quality conservation work’ (P15), ‘having the opportunity to conduct research that has been co-created by a wide range of stakeholders’ (P13), opportunity to learn ‘a lot about beavers and their ecology’ (P14), and being ‘better informed and prepared to adapt our own strategies and operations, and advise others in future catchment management approaches’ (P7). This suggests that, if stakeholder engagement is effective in future projects, stakeholders may find participation in future projects to be of value also. All participants indicated a willingness to participate in future reintroduction trials, particularly ‘if [the] species was relevant to/impacted on’ (P12) their respective interests. P14 said they would take part ‘to study and learn more and offer advice if helpful’. However, P9’s willingness to participate was conditional ‘provided the various criteria for objective trials […] are met’.

### Research and monitoring programme

Reintroduction trials require a well-planned scientific research and monitoring programme to meet project objectives and IUCN Guidelines (Ewen et al. 2014; IUCN and SSC, 2013). Broadly, the ROBT’s monitoring and research programme was viewed as a success; when asked about successes, twelve participants cited the science and/or monitoring programme, or the *Science and Evidence Report* (Brazier et al. 2020a). E.g. P16 stated ‘I think the ultimate success of the trial has been the high quality of research that has been conducted. It has provided an evidence base for decision-making and highlighted areas where more work is needed’.

In accordance with stakeholder engagement as discussed above, we suggest the research and monitoring programme should be co-created with stakeholders. Indeed, P13 said ‘Having the opportunity to conduct research that has been co-created by a wide range of stakeholders has been a very positive experience’. By engaging stakeholders, questions or concerns are more likely to be addressed early. Greater stakeholder trust in the research may reduce conflict potential, and early engagement during design stages may facilitate such trust between stakeholders and researchers (Riley et al. 2018).

Demonstrative objectivity will also facilitate trust. Here, P9 questioned the objectivity of research and felt there was ‘bias towards beaver monitoring but poor collection of evidence on impacts on fisheries’. P1 (a researcher) suggested scientific peer review may be one avenue through which a demonstration of objectivity and rigour could be achieved. When asked about risks/challenges of trial participation, they said ‘Maintaining scientific integrity and impartiality. Whilst our role as University researchers is to undertake independent research, our position as the main project partner has at times led some to question this. Have sought to address via peer review of results etc.’.

With co-creation in mind, the research focus will need to give a ‘holistic understanding’ (P13) of a reintroduction, beyond single interests of contributors. This is for the social and ecological consequences of a project to be sufficiently understood, as recommended by IUCN Guidelines. Additionally, the programme may need to be reactive to emerging issues and changing circumstances: ‘Balancing the need for a clear programme of research with the value of being able to be reactive so research can focus on areas that emerge as being of key importance or lacking in existing evidence’ (P16).

However, there can be limitations. Three were evident in our findings, which are reported in Table 3. Limitations may lead to outstanding research questions, as P16 suggested: ‘it will not be possible to answer all the questions within the scope of such trials’. For example, there were outstanding research questions at the ROBT’s conclusion about the relationship between beavers and fish: ‘it is a shame that there couldn't have been a more definitive conclusion on the impact of beavers on migratory fish populations, which appears to remain as one of the points of contention. Although the Trial provided some good evidence on this issue, the work wasn't sufficiently comprehensive or of sufficient duration to enable a clear conclusion and consensus to be achieved’ (P2). It should be noted that where important questions remain unanswered, uncertainty may prevail. Uncertainty can lead to increased worry, making it likely that concerns escalate (Auster et al. 2020b; Hudenko 2012). For example, P16 said ‘Those with particular interests in these topics may well feel that a lack of information = failure’. P16 then suggested ‘What is important is that research continues where it is needed (and that this is well resourced)’. We suggest in the initial stages of reintroduction (alongside research planning) stakeholder expectations need to be managed regarding research feasibility, and stakeholders themselves may need to assess their expectations of what is feasible within the project scope and limits. If the reintroduced species is to remain in the longer term, addressing uncertainty with ongoing research into outstanding questions may help reduce worry and reduce conflict potential, particularly when associated with management that can adapt to emerging evidence (Hudenko 2012; McCarthy and Possingham 2007). Here, research into the relationship between beavers and fish (particularly fish migration) is likely needed to continue with open, cross-sectoral dialogue throughout (Auster et al. 2020a): ‘I sense this is an area for further work and dialogue’ (P7).

**Table 2:** Challenges in stakeholder engagement that were reported by participants

Challenge	Description of the challenge	Example quotes
Participation from stakeholders	Despite outreach effort, some stakeholders may not fully engage	‘there were a few organisations and individuals that didn't participate - despite being invited and wanting to be involved’ (P4) ‘It is disappointing that some groups who have raised concerns regarding the trial were invited to sit on groups but chose not to participate’ (P10)
Risk of partnership breakdown	Risk of unresolvable conflict between groups (N.B. No participants reported a breakdown in the ROBT)	‘[Risk in] partnership interactions and potential breakdown.’ (P6) ‘...adopting such a [...] stance risks alienating some stakeholder groups’
Reputational risk	Risk for stakeholders that engaging in a project may influence perceptions of the stakeholder	‘[Risk of] the public automatically thinking we are anti-beaver because we are a landowner.’ (P19) ‘The risk of being seen to be 'pro-beaver' rather than having objective views based on empirical evidence.’ (P9)
Potential use of stakeholder resources	Risk that participating would require high levels of input	‘There was an initial risk that our staff may need to devote considerable time to working with the Project Team and affected land owners [...] However, in the event, there was only a very limited need for such input during the Trial.’ (P2) ‘Involvement needs to be adequately resourced.’ (P16)

**Table 3:** Limitations on the research programme that were reported by participants

Limitation	Description of the challenge	Example quotes
Financial resource	Balancing the desired level of research with the amount of funding that is available for it	‘It has been challenging to undertake the wide range of research that stakeholders have demanded during the trial and particularly challenging to secure enough funding to deliver all aspects of the research program that were asked for by stakeholders, including other members of the steering group’. (P13) ‘…research across 5 years on a trial of this scale is very time consuming and very costly’. (P13) ‘With further funding there is of course additional research that would have been extremely valuable to undertake. However, even with the massive efforts […] to raise money it was still necessary to prioritise certain aspects of the research’. (P10)
Practical limitation	Ability to research beaver impacts where there are limited examples of the impact itself, or where beaver behaviour is unpredictable and varied	‘Monitoring beavers is very challenging due to the spatially and temporally variable nature of their impacts. Designing suitable monitoring frameworks can therefore be challenging and some studies had to be altered or abandoned due to changes to beaver activity. Therefore, not all of the desired investigations were completed’. (P10) ‘a possible limitation of the project was (ironically) the lack of more problems. Although the beavers did create 'issues' in a number of locations which required active management to mitigate the potential consequences, which proved to be extremely instructive element of the Trial, it might have been better if there had been even more of these types of localised problem’. (P2)
Temporal limitation	Capacity to address research questions that may require time before they can be answered	‘Many of the positive and negative impacts of beavers would not be seen until population numbers reach (initially overshoot) [ecological] carrying capacity’ (P14) ‘It has been a trial of the early phases of beavers recolonising a catchment’. (P15) ‘a challenge to work with certain stakeholders who either did not engage in the learning process at all or did so very late on in the trial, thus not leaving enough time to undertake research to answer their questions’ (P13)

In addition, P18 felt future projects should ‘learn from other projects/experiences to build on knowledge, don’t reinvent the wheel, take more things as red with confidence ‘(P18). This suggests that research could build upon prior knowledge, rather than cover topics addressed elsewhere. Indeed, P8 said in the ROBT it ‘Sometimes felt like lessons and experience/expertise from Scotland were not being fully taken into account or utilised and potentially, therefore, re-inventing the wheel when this was not needed’.

### Strategy to manage arising conflicts

Reintroduction projects should anticipate and seek to prevent or manage conflict issues, with proactive action likely to be received well (Auster et al. 2020b; Auster et al. 2020d; IUCN and SSC 2013; Sutton 2015). Reflecting on this, our results indicated a need for projects to have a management plan for conflict scenarios. When asked about lessons for future reintroductions of beavers or other animals, twelve respondents referred to the ‘importance of a management framework’ (P7). Here, this refers to the management of beavers and conflicts (as opposed to project management or research monitoring). E.g. P15 said ‘Do not leave landowners to cope with reintroduced species on their own. Provide support’, and P1 stated ‘Most conflict or perceived conflict can be managed, but this does require a clear management plan’.

Such a plan will require appropriate engagement with individuals who experience negative impacts (Auster et al. 2020b). In the ROBT, DWT held responsibility for management in accordance with the licence. It is important to recognise this incurred resource use for management measures (Brazier et al. 2020a). Future projects should be equally prepared to address conflict situations; ‘Resources (personnel, time, money) need to be allocated for managing potential conflicts’ (P16).

Ideally, management actions would be undertaken proactively, addressing issues prior to occurrence (Auster et al. 2020d). In the ROBT, P12 stated they felt ‘proactive action […] was successful in this case’. This may be impossible for all potential conflict issues, in which case, it is desirable to address issues quickly to minimise or prevent conflict escalation (Auster et al. 2020b; Seddon et al. 2007); P2 cited a trial success in ‘The establishment and maintenance of a strong mitigation strategy, to deal with issues quickly and effectively as they arose’. This comment is supported by a previous study which interviewed individuals who reported conflicts with beavers in the ROBT; proactive action and fast responses were highlighted as positive among participants (Auster et al. 2020b).

Clear communication of a management plan can bolster community knowledge of available support. This may reduce conflict potential by providing certainty in management (Auster et al. 2020b). P10 said ‘communication with all effected stakeholders and (as many as possible) landowners is key’. They stipulated ‘If people know they can call on somebody to help if there are issues they are much more willing to take part and learn from the experience. In almost all cases, this was done extremely well during the ROBT’. Linked to this, project leads and those responsible for communication may need to consider their positionality when outlining available management support. Here, P11 said ‘this has been led and managed from an organisation with a very particular slant […] linked to this has been the funding requirement. This has meant publicity and campaigns that have been pejorative and requiring the development of “beaver connection”’. The respondent felt ‘this makes the conversation quite led and perhaps difficult with regard to “selling” the need for parts of the management hierarchy’.

When a species is to be reintroduced permanently, long-term thinking should structure the management strategy; the effectiveness of techniques together with attitudes to management should be considered (Auster et al. 2020d). In the ROBT, there was uncertainty among stakeholders about the management of negative beaver impacts in the long term. This increased uncertainty led to increased levels of worry (Auster et al. 2020b; Hudenko 2012). For instance, P19 said ‘We remain nervous as to the degree to which there will be support for beaver management post Trial’. As above, not all long-term scenarios can be predicted so, as with coexistence of other species, we suggest adaptability must be included in management considerations (Failing et al. 2013; McCarthy and Possingham 2007); P16 stated ‘What is important is that […] the management of beavers is adaptive to the new evidence that emerges’.

Some long-term decisions may not be in the hands of project leads or stakeholders but with government or local authorities, e.g. decisions on the application of legislation. It is nonetheless possible for practitioners and stakeholders involved in projects to collaborate, share learning from experience, and provide informed recommendations. For example, in the ROBT a ‘management plan [was] co-created by a broad spectrum of project partners’ (P1). These partners sat on the BMSF, formed by the SG (Table 1). The plan they developed—the ‘Post-2020 Beaver Management Strategy Framework’ (River Otter Beaver Trial 2019)—was cited as a success by four participants. Collaboration and knowledge-sharing like this could occur across projects. P8 highlighted a need for ‘consistency across projects’. For instance ‘each project doesn’t need to reinvent the wheel’ as—if the species remains permanently—‘there will also be a need to have a national approach […] as animals transition into just ‘being there’.

Additionally, ‘the risk with anything new is that management systems and processes are overly complex and intensive’ (P6) when there is a desire for management to be accessible. Indeed, in a previous nationwide questionnaire, several respondents indicated stronger levels of legal protection may make management of negative beaver impacts more difficult, (Auster et al. 2020d; Brazier et al. 2020a, p81). This will also require consideration as to how to ‘normalise the species’ (P18) in a landscape (Auster et al. 2020b): ‘although there is always room and need for more science and learning, any such project in the future […] has to be more orientated around management advice and interventions (where necessary) to help ensure as smooth a transition from beavers being seen as new to the landscape, to the wild, to the way rivers work, to a position where beavers are seen as being a natural part of all that’ (P7).

Finally, it is important to remember that one of the reported stakeholder engagement challenges (Table 2) was the willingness to participate of some stakeholders. This could have bearing on the effectiveness of management plans in the future; if engaging stakeholders early is more likely to reduce potential for conflict escalation, then it is reasonable to assume that where stakeholders opt to not participate, issues could continue to arise later if these groups feel their concerns have not been addressed (Auster et al. 2020d; Coz and Young 2020). In this event, we suggest a continued openness and willingness to engage in constructive discussion (alongside the ability for management strategies to be adaptive (Failing et al. 2013; McCarthy and Possingham 2007)) will be likely to facilitate better integration of additional perspectives into management frameworks later. However, as this is a discussion that looks beyond the timeframe of the River Otter Beaver Trial, this will be an area for further research. We recommend continued investigation into how stakeholders continue to act or respond as reintroductions move beyond their initial stages, as well as further study into the best approaches of incorporating new stakeholder perspectives into adaptive management frameworks as the presence of the reintroduced species is consolidated.

### Public engagement

Public engagement is critical in species reintroductions (IUCN and SSC 2013), and ‘social buy-in’ is important if a reintroduction is to be successful (Hiroyasu et al. 2019). In the ROBT, P1 claimed there was a ‘high degree of public engagement and support’, and P4 said ‘doing lots of outreach work has been vitally important’.

There were several reasons cited as to why. First, an opportunity to educate the public and address misunderstandings: ‘a forum for clearing up issues as miss understandings [sic]’ (P12). This includes potential for education via the press, which P15 cited as a ROBT success: ‘generating press interest and articles that show beavers can play a role in creating more flood resilient landscapes and create habitats for a wide range of biodiversity’. This may be important for garnering community support, with engagement leading to education and influenced attitudes (Hiroyasu et al. 2019; Sampson et al. 2020).

The tone and framing of public engagement must be considered. Niemiec et al. (2020b) suggested that presenting extreme positive arguments, whilst not addressing concerns of opponents, is likely to lead to organised opposition. They suggest message framing should be more moderate. In the ROBT, and despite their organisation’s stance in favour of beaver reintroduction, P4 felt a balanced approach to their engagement work had been beneficial: ‘Our presentations are balanced rather than overly positive, which I think has helped’. This included ‘being open and honest about the conflicts that beavers can cause’ (P4). P9 however felt their own ‘main success has been to expose the issue of beaver re-introductions to a wider audience, offset[ting] the overwhelming pro-beaver position of most participants’.

Alongside ongoing research, public engagement should involve provision of knowledge surrounding management support to reduce uncertainty and address concerns (Auster et al. 2020b; Hiroyasu et al. 2019; Niemiec et al. 2020a, b). P15 said ‘Many landowners may be against reintroductions but attitudes can change over time if those landowners feel involved in the trial and feel that they […] have support if required’ (P15). In the longer term, such messaging may facilitate coexistence with reintroduced species; P6 cited ‘Community engagement enabling people to learn to live alongside the animals once again’ as a ROBT success.

### Broad perspectives on reintroduction trials

Survey participants also provided broader comments on the process of reintroduction ‘trials’. For some, the ROBT was perceived as a model to follow in future. For example, P10 said ‘I believe the ROBT provides an excellent framework on which to design reintroduction programmes’ and P14 felt the process provided ‘evidence and understanding on whether reintroductions of a particular species could or should take place, and if so, how they should be carried out’ (P14).

Although participants were generally favourable of the principle of a reintroduction trial, further lessons could be learned. P11 believed ‘[reintroduction trials] need to be developed but this was a very important first start’. Nonetheless (as discussed), there will still be room for more learning at the end of a trial phase. For example, P16 stated ‘trials such as this are invaluable for providing context-specific evidence. The caveat is that it will not be possible to answer all the questions within the scope of such trials. The end of these kinds of trials does not indicate that no more research is required’.

Some participants had issue with how the trial began. P12 noted the beavers were already present prior to commencement and felt it’s not really a model for re-introduction as [it is] a model for how to deal with escaped and feral animals. If looking for how to do a introduction properly from the start it would not be the recommended approach to let the animal loose and then deal with it’. This was cited by P5: ‘the dubious nature of how the beavers arrived is always a bone of contention amongst farmers’. This is a notion that was also reported in Scotland resulting from the appearance of a population of unlicensed beavers in Tayside; the lack of formal process in the reintroduction was observed to be the main driver of post-reintroduction conflict, leading to a lack of trust between stakeholders (Coz and Young 2020). Recognising the unplanned nature of introduction in Devon, Crowley et al. (2017b) suggested the ROBT was an opportunity for a ‘wild experiment’, gaining experience in managing issues and ‘finding ways to include affected and interested publics’.

Although reintroduction trials were broadly supported here, future trials may not need to echo the same scale for other species, particularly those that do not have landscape-scale impacts. P15 said ‘For other species a trial reintroduction could be useful, other species may not require such work as they may have less of an impact of surrounding landscapes’. Indeed, P2 felt ‘beavers are not like most other species which are subject to reintroduction programmes’. This is due to the scale of landscape change attributable to beavers; ‘other species may not require such work as they may have less of an impact of surrounding landscapes’ (P15).

Respondents suggested population sizes may limit the ability to collect necessary evidence to meet trial objectives. Thus, depending on objectives defined at the outset, some research may require time before a larger population can exist (see Table 2), thus allowing for certain impacts to materialise and be studied. Accordingly, P18 believed a trial should have ‘greater aspirations on numbers and scale’. However, P11 and P9, who held more concerns about the reintroduction, felt there needed to be a cautious approach with a ‘clearly articulated ‘out’ at the start of the project’ (P11) in case of negative consequences. Whilst a bigger population may enable further research, in the event of a decision not to formally reintroduce the species at a trial’s conclusion, removal of the species from the landscape may become more challenging. This ability may be key in the engagement of some opposition groups; P9 stated they would participate in future reintroduction trials ‘Only if the trials are truly objective and capable of being ended/reversed in the event of potential adverse impacts’. Consequently, there is likely to be trade-off required and, again, expectation management of research feasibility among involved parties. (Additionally, related to population size, P18 felt that there was a trial failure in ‘not establishing a genetically diverse population’. Genetic diversity should be factored into population establishment (Campbell-Palmer et al. 2020; Halley 2011)).

Similarly, decisions are needed on the duration of any trial. P16 referenced ‘there is scope for [research] to continue for decades’. If this were to occur, however, this would be resource-intensive and potentially delay any decision on species reintroduction indefinitely. Again, therefore, we suggest that there will need to be some trade-off, with a decision taken on what trial duration is necessary, with ‘realistic expectations of what can be achieved during a relatively short trial reintroduction (perhaps particularly the expectations of various stakeholders)’ (P16). Therefore, as discussed above, research may need to continue beyond the end of any trial, but trials may ‘provide a starting point for decision making and management and this should be made clear to all stakeholders involved’ (P16).

Finally, some participants, although favourable towards trials, felt that they risk resource-intensive processes which inhibit the potential of reintroductions. P18 believed reintroduction trials should ‘not be overly cautious […] This type of trial set-up has a time and place but […] we are in danger this sets a precedence that conservation translocations require this level of investment every time, when other similar sectors have no such requirement or expectations thereof […] we need to be careful we aren’t holding up reintroductions as overly complicated and expensive and therefore subject to continued scrutiny’. P6 agreed: ‘We are facing a climate and ecological crisis—species reintroductions need to be done well—but the barriers organisations face are often prohibitive and costly’.

## Discussion

We have identified a series of themes from the points of view of stakeholders involved in steering a reintroduction project. In this section, we will discuss how this relates to the previous study and examine what is different in reintroductions for conflict and coexistence issues. Prior to doing so, however, we would like to advocate for reflective evaluation to be an essential part of future reintroductions. All reintroduction projects provide opportunities to learn from the process undertaken. Such learning may inform and improve the steering and expectations of future reintroduction projects. As reintroductions are growing in popularity and uptake increases (Corlett 2016), many participating groups will likely be ‘learning-as-they-go’. Here, we undertook a reflective evaluation of the ROBT with key informants and suggest the points made under the identified themes will prove informative in future reintroduction projects—for practitioners, stakeholders, and researchers alike. Undertaking evaluations such as these within other projects would enable knowledge gained through experience to be shared, affording the opportunity to apply knowledge in future project contexts. We would encourage this as a standard practice, including both when projects succeed and fail (Catalano et al. 2019).

In this analysis, there are points identified which reinforce the findings of research from pre-existing conflict and coexistence literature but in the reintroduction context, particularly about stakeholder engagement. In 2016, Decker et al. outlined ten ‘governance principles for wildlife conservation in the twenty-first century’. These include the incorporation of multiple, diverse perspectives in governance, as well as governance that is transparent and accountable. This is reflected in the participants’ assessments of the ROBT, with thirteen participants citing stakeholder engagement as a key trial success, but with a perception among some that group leadership should have been independent to facilitate transparency. This may help build trust between parties; trust building is essential for the resolution of conflict issues, which can be built through demonstrable efforts to recognise and respond to issues (Madden 2004). Riley et al. (2018) examined trust in wildlife agencies and identified that trust was greater when personnel actions created a sense of fairness for stakeholder involvement. Extremely positive (or negative) messaging meanwhile may have the opposite effect, with a risk of more organised opposition and resulting conflict escalation (Cusack et al. 2021; Niemiec et al. 2020a, b). There may always be issues affecting trust (Riley et al. 2018), but investment in trust-building and incorporating stakeholder viewpoints will mean it is more likely that conflicts can be negated and coexistence achieved for both reintroduced and already present species (Bennett et al. 2017; Coz and Young 2020; Redpath et al. 2015; Riley et al. 2018).

As stated above, the increasing uptake of reintroduction projects means many practitioners are likely to be ‘learning-as-they-go’. We believe that the relatability between our findings and previous study can provide practitioners and stakeholders with reassurance that preexisting knowledge from non-reintroduction-related experiences is applicable also within this emerging field. That said, by eliciting the views of the ROBT Steering Group stakeholders in a way that was meaningful for them, we have identified an important distinction between reintroduction and pre-existing research, regarding conflicts and coexistence.

Coexistence with a reintroduced species is a specific form of coexistence as humans in the locality are likely to have no prior experience of the historically present species. Hence, coexistence challenges in reintroduction begin from a different start-point; projects seeking to facilitate coexistence in response to conflict issues start from the point at which the issue exists in the present, whereas in reintroductions potential coexistence challenges are in the future (post-reintroduction). In our participants’ responses, this sense of the future context is evident. Under theme 3, for example, participants identified outstanding research questions at the trial end, particularly regarding interactions between beavers and fish. Research questions which remain unanswered can lead to a sense of uncertainty which in turn can lead to worry or concern about future consequences, influencing decision-making (Hudenko 2012). In reintroductions, the decisions to be influenced by uncertainty can go beyond how best to coexist with a species to include whether or not to coexist with it in future at all, meaning uncertainty could trigger coexistence to be delayed or prevented altogether.

When a reintroduction does take place, theme 4 identified the importance of a planned management strategy to respond to conflicts if coexistence is to be achieved. Where management is developed to facilitate coexistence with present species they are reactive to challenges that exist and are able to engage with stakeholders who hold experience of those challenges (Clark et al. 2016; Frank 2015; König et al. 2020; Madden 2004). In reintroduction, conflicts with the species can only exist after the species is released. The participants here demonstrated concerns about the ability to address potential conflict issues and whether management would be ‘bound up in red tape’ (P17). Such questions perhaps represent a fear of unknown future consequences, with uncertainty again contributing to concern. This represents the need for practitioners to engage with stakeholders at an early stage of a reintroduction project and consider potential options for management a priori (Auster et al. 2020d; Coz and Young 2020; Seddon et al. 2007). In this instance, the aforementioned Beaver Management Strategy Framework developed within the ROBT (River Otter Beaver Trial 2019) was arguably an example of such a forward-thinking approach within the early stages of a reintroduction; it was developed between stakeholders to consider the management of the River Otter beavers post-2020, but in advance of the UK government decision to allow them to remain permanently from that year.

To encourage such future-orientated coexistence thinking in future reintroduction projects and research, we argue for the definition of a new term: ‘renewed coexistence’. We define this as coexistence that is specifically associated with a reintroduced species, thereby one which was present in the landscape historically, but which will likely be a ‘new’ presence for the humans living in the locality post-release. By building on the term ‘coexistence’, the new term recognises that it is built upon pre-existing knowledge that coexistence is adaptive and dynamic to be sustainable, with conflict management where required (Carter and Linnell 2016; Frank 2015; König et al. 2020). With the application of ‘renewed’, the term recognises the ‘newness’ of the presence of the formerly resident species for humans in the landscape today, thus allowing for an appreciation of questions unique to the context as humans learn to live with the reintroduced species (such as that discussed under theme 4 about how to normalise the sense for people that the species is a wild rather than reintroduced animal).

We argue that our definition of ‘renewed coexistence’ (as a branch of coexistence study) will provide the necessary emphasis for groups steering reintroduction projects to consider future coexistence challenges in this specific context, engaging with affected stakeholders early to address uncertainty, and encouraging an a priori attention towards the management of potential future conflicts to achieve coexistence with reintroduced species, if and where reintroductions occur (Auster et al. 2020d; Coz and Young 2020; Seddon et al. 2007).

## Concluding remarks

Reintroductions seek to establish a population of formerly resident species and garner benefits, such as the restoration of ecosystems or their functioning. By their nature, they are projects that think into the future and have implications for the long-term. Reflecting on an analysis of the experiences of stakeholders involved in steering a reintroduction project, we coined a new term to advocate for the consideration of future coexistence issues in a similar vein: ‘renewed coexistence’. We trust our new term will encourage early and forward-thinking approaches to coexistence with reintroduced species, addressing potential conflicts a priori and reducing uncertainty. As advocated for in pre-existing coexistence literature, we believe that ‘renewed coexistence’ is more likely to be achieved and sustained with effective project governance and early stakeholder engagement (Auster et al. 2020d; Coz and Young 2020; Treves and Santiago-Ávila 2020; Zimmerman et al. 2020; Seddon et al. 2007). Finally, when reintroductions do take place, we believe this style of forward-thinking would lead to more effective conflict management and facilitate better integration of reintroduced species into anthropogenic landscapes.

### Electronic supplementary material

Below is the link to the electronic supplementary material.

Supplementary file1 (DOCX 103 KB)

Supplementary file2 (DOCX 26 KB)

Supplementary file3 (DOCX 43 KB)

Supplementary file4 (DOCX 264 KB)

## Data Availability

All data generated or analysed during this study are included in this published article (and its Supplementary Information files). The anonymised survey data is available in full (with participants’ details redacted) in the supplementary information.

## Appendix 1 – Participant Stances and Motivations

Summary of participant stances held on beaver reintroduction (in their own words), their motivations to participate in the ROBT (in their own words), and the groups upon which they sat.

**Table:**

Participant	Stance on beaver reintroduction	Motivation to participate in the ROBT	Group(s) sat on		
			SG	BMSF	S&E
1	“To undertake impartial research to understand the impacts of the return of beaver to great Britain and inform policy.”	“Research”			✔
2	“My organisation has expressed conditional support for the project, but does not have a clearly established position on beaver reintroduction.”	My organisation has a range of interests / statutory functions that had the potential to be directly affected by the presence of beavers within the catchment. […]. On this basis […] our organisation had highlighted practical issues that we felt needed to be satisfactorily addressed through it”	✔		
3	“…initially uncertain about beaver reintroduction (some years before River Otter beavers became public knowledge). As a result of considerable advocacy work by both in-house ecologists, and independent advisers […] we took up a stronger positive outlook and adopted and initiated two beaver "reintroductions" (both within enclosed pens) […] These were enthusiastically supported by most of staff (though not all […]). […] [Organisation] now I think a strong supporter and advocate, provided done with all regard to	“I was very keen to ensure two things: One was that the practical experience of lethal control and humane trapping (and deporting or humane despatch) of wildlife from our Wildlife Ranger team […] was made available to the ROBT for development of the "post release" management of beavers once they become abundant across the UK landscape. [...] secondly I was very keen for [organisation] to both be involved and to be seen internally to be involved, thus promoting both the role of the rangers in a wider conservation programme of considerable import and excitement, and to bring the knowledge and experience of the ROBT to bear on [organisation’s] landscapes.”	✔		
	legal and scientific protocols adhered to, and perhaps, as importantly, seen as acceptable to neighbouring landowners and their interests.”				
4	“promoting the reintroduction of beavers back into Britain.”	“Huge range of benefits in terms of ecosystems, natural processes, ecosystem services. Beavers are a keystone species, without which our ecosystems are in poorer condition.”	✔	✔	✔
5	“None”	“Represent the farming community and learn. Thus providing more rounded info back to the farming community.”		✔	
6	“We wish to see an ambitious strategy for beaver reintroduction throughout Devon and the UK.	“Project Lead. Recognition of the keystone role beavers will play in the future health of our ecosystems.”	✔	✔	✔
7	In the broadest sense, we are aware that there are opportunities and risks associated with the return of beavers to rivers in England. We have been	“To support and learn from a project that has been all about trying to better understand the opportunities and risks associated with beavers in the wild in England.”	✔		
	working and continue to work with other organisations and projects such as the River Otter Beaver Trial, to better understand these opportunities and				
	risks, help us inform and advise defra on their future policy on beavers, and be best prepared to deliver and support any management measures that may be required by us and others.”				
8	“We have a "yes if" approach with an informal approach document explaining the [organisation’s] roles and remit. This stance has remained the same and the trial has helped inform further.”	“[Organisation has] a significant role in the water environment operationally, advisory and regulatory. Presence of beavers can influence all of these in varying aspects of our work and also potentially deliver a number of objectives in a positive way, in particular around working with natural processes. There are also aspects where presence may bring challenges and so the trial provides/provided was/is useful and informative.”	✔	✔	✔
9	“Our concern is with the potential impact of beaver introductions on threatened migratory salmonid populations, principally on blocking migration routed to	“Long-term conservation of salmonids and the socio-economically valuable angling they support”	✔	✔	
	and from spawning areas and replacement of flowing streams suited to juvenile salmonids with slow areas not suited and liable to temperature increases”				
10	“… to undertake research to inform of the impact of beaver reintroduction and to understand the positive impacts of beaver on the landscape alongside the management challenges that they present. This has remained consistent throughout the trial”	“Personally, to undertake research as part of my PhD research which was twinned with the trial. As an organisation, our motivation has been to gain greater understanding of beaver impacts in a landscape dominated by intensive landuse.”			✔
11	“yes we do [hold a stance] and I don’t think it has [changed over the course of the Trial]. the stance is in part based on govt support and the legislative framework for which we still lack clarity on either”	“we represent the landowners and farmers on whose land beavers will provide a benefit and impact”	✔	✔	
12	“interested Observer- remains unchanged until legal status is decided”	“possible value of beaver as a tool for catchment management”	✔		
13	“As a scientist I am impartial about Beaver reintroduction. As a research institute, the [organisation] also adopts an impartial position.”	“To undertake research, build understanding and knowledge and disseminate this knowledge to a wide range of stakeholders.”	✔	✔	✔
14	“Professionally, and without affiliation to any organisation in favour or against the reintroduction of beavers, I have always taken a neutral stance and have principally been interested in the science.”	“As a scientist who has studied the behaviour, ecology and welfare of mammals, especially rodents, for many years I was attracted to the challenge of studying and learning about a potential newcomer to England, the beaver. The motivation has always been to understand the science of beaver reintroduction, the challenges of getting good data and their interpretation, i.e. evidence based conservation.”	✔		
15	“Pro beaver reintroduction. This has not changed over the trial.”	“[Organisation] one of the main partners”	✔		✔
16	“I can't speak for others in the organisation, but personally, I was very pro-beaver reintroduction to England prior to involvement in the ROBT. I would say I still am, although feel I have a greater appreciation of the challenges and conflicts that beaver reintroduction can have. While I believe that, on balance, the overall benefits associated with reintroducing beavers out weight the costs, I am more understanding to the fact that in some locations / scenarios (i.e. at smaller more site specific scales) this may not always be the case.”	“I was personally motivated because it represented an opportunity to conduct field work on a really exciting ecological / conservation topic and one that was of genuine interest to me. It also provided an opportunity to work alongside other research groups […] and organisations […], which I enjoy. From an organisation perspective, I suspect the reasons are similar, but also include the fact it related to existing research that was being conducted within the group, aligned with our areas of expertise and was an opportunity to continue research on the topic.”			✔
17	“[Organisation] welcomes reintroductions of native species provided they follow IUCN guidelines. We do, however, take a slightly cautious approach because we believe that reintroductions often make light of the mitigation and control issues that are likely to arise if they are successful. None of this has changed as a result of ROBT”	“Believeing that beaver reintroducion would be successful, we were keen to try to ensure that future management and protection status would be as simple as possible. We were also keen to ensure that proper account was taken if likely negative impacts, especially on migratory fish.”	✔	✔	
18	“Pro-beaver reintroduction, hasn’t changed [over course of the Trial]”	“Working directly with beaver reintroduction and research for the last 12 years in Scotland and wish to see the species officially and responsibly restored across Britain.”	✔	✔	
19	“Our stance has remained unchanged. Cautiously supportive as long as management considerations are addressed. We feel they have been as much as the Trial will allow. We are obviously better informed than at the start, but to be honest weren't poorly informed to begin with. It has largely played out as we expected.”	“Influence the outcome - particularly related to management approach. We saw early on that beavers were in England and expanding and that there was no political will to ever remove them. Thus, in our view better to accept this and be on the inside influencing the process to get the best outcomes for beavers and land managers than grumbling from the sidelines.”		✔	✔

## Appendix 2: Additional Beaver-Specific Points

Additional points that are unique to beaver reintroduction following the ROBT were made by some respondents, beyond the six overarching identified themes. These are summarised in the table below. To note, these points may not necessarily reflect a view held unanimously by all participants.

For a comprehensive account of the science and evidence from the ROBT, please see the *River Otter Beaver Trial Science and Evidence Report* (Brazier et al., 2020a).

**Table:**

**Additional Beaver-Specific Points**	**Example Quotes**
Beavers will survive in Britain	"beavers populations can thrive in our landscapes." (P1)
Beavers are net beneficial	"there is strong evidence of the benefits which derive from the presence of beavers in a catchment (environmental and, potentially, economic)" (P2)
	"Results from early stages of beaver catchment colonisation indicate overall environmental impacts are positive." (P1)
	"Many potential future reintroductions could create measurable benefits to our environment and wider society. Exeter uni work is demonstrating this is the case for beavers. (P15)
There is a need for resourced management and support for individuals who experience negative beaver impacts. This may require dedicated beaver officers	"The trial, for the first time in England shows that the benefits of beaver can coexist alongside appropriate management of undesirable impacts.” (P10)
	"Its far too costly and complex an approach to be adopted or advocated in other catchments...it needs to be seen as a proxy for other catchments and not a model." (P3)
	"Professional "beaver officers" are likely to be a key to successful integration of beavers into wider catchments." (P3)
There is no more need for beaver trials on the same scale.	"I think that the weight of science and evidence, the coherent management strategy and the conclusions of the trial suggest that running such an in-depth trial again would not be necessary. Pretty well all of the findings transfer to the vast majority of English catchments, what is needed now is a well-funded management framework for the reintroduction of beavers, based on the outcomes of the ROBT." (P13)
	"For beaver in GB it should not be necessary to go to the lengths that the ROBT went to now that the findings have been collected. The cost and time frame of similar trials would provide only limited additional information whilst slowing progress in terms of both beaver managment and introduction. That being said, I do believe that future introductions of beaver, if they are to go ahead, should require some level of monitoring and funding to ensure that appropriate management and understanding is in place" (P10)
There is room for ongoing research to plug knowledge gaps, in particular the interactions between beaver activity and fish migration.	[Referring to key Trial failures] “"Not resolving deep seated beliefs regarding beaver and fish interactions" (P6)
	“I believe there remain still, some very strong views and concerns, particularly from fisheries interests about the risks to fish passage and fish habitats from beaver activity. The project has contributed important information to this debate. However, there will only be so far that this could have been taken forward, in what has been a relatively short period, in the context of what beaver activity there has been, and dependent on other factors during this project. I sense this is an area for further work and dialogue, as we go forward." (P7)
	“Although wrong to characterise it as a failure, it is a shame that there couldn't have been a more definitive conclusion on the impact of beavers on migratory fish populations, which appears to remain as one of the points of contention. Although the Trial provided some good evidence on this issue, the work wasn't sufficiently comprehensive or of sufficient duration to enable a clear conclusion and consensus to be achieved." (P2)
	[Referring to key Trial failures) "poor collection of evidence on impacts on fisheries" (P9)
Accurate information about beavers needs to be widely disseminated.	“Public, institutional and private understanding of the true nature of beavers, and the impact they may have (both beneficial and negative), needs to be both professional and accurate, and widely disseminated to a wide range of stakeholders and interested parties." (P3)
