# Supplementary material for: Renewed coexistence: learning from steering group stakeholders on a beaver reintroduction project in England
Source: Eur J Wildl Res. 2021 Dec 3;68(1):1. doi: 10.1007/s10344-021-01555-6 (PMC8640482; doi:10.1007/s10344-021-01555-6)
Supplement: Supplementary file 1 — Supplementary file1 (DOCX 103 KB) [file 10344_2021_1555_MOESM1_ESM.docx]

# SUPPlementary INFORMATION: DATA

**Article**: Renewed Coexistence: Learning from Steering Group Stakeholders on a Beaver Reintroduction Project in England

**Journal**: European Journal of Wildlife Research

**Authors**: Roger E Auster (University of Exeter; [r.e.auster@exeter.ac.uk](mailto:r.e.auster@exeter.ac.uk)), Prof. Stewart Barr (University of Exeter), Prof. Richard Brazier (University of Exeter)

## A reintroduction trial ‘on trial’

This document provides the data from survey responses. Any personal information (or identifiable information) has been redacted. This includes removal of all responses to ‘Section 1: Your Details’.

To note, the following statement was provided for participants about the sharing of data in the research information:

“Data will be stored securely and anonymously at the University of Exeter and then shared in a public repository in order to make it available to other researchers in line with current data sharing practices. The shared data will not include any personally identifiable information. If you have directly attributed any answers to your organisation, these comments will be redacted unless permission has been granted by yourself for them to be included.”

If a participant took the opportunity to comment from the Findings Report (also available as Supporting Information), their responses are also provided here. Participants were informed their responses would be subject to all the same research and data protection considerations as survey responses. Participants were asked to give permission for their Findings Report responses to be quoted.

## PARTICIPANT 1 (P1)

### SECTION 2: Your involvement in the Trial

The next three questions will ask you about which group(s) you sat on. For each one that you indicate, you will then be asked to indicate your role upon that group from a check-list.

#### 1. Were you a member of the ROBT Steering Group (SG)?

No

#### Please use the following checklist to indicate your role(s) upon the Steering Group (SG). You may select as many answers as are appropriate.

*This question was not displayed to the respondent*.

#### 2. Were you a member of the ROBT Beaver Management Strategy Framework Working Group (BMSF)?

No

#### Please use the following checklist to indicate your role(s) upon the ROBT Beaver Management Strategy Framework Working Group (BMSF).You may select as many answers as are appropriate.

*This question was not displayed to the respondent*.

#### 3. Were you a member of the ROBT Science & Evidence Forum?

Yes

#### Please use the following checklist to indicate your role(s) upon the ROBT Science & Evidence Forum (S&E).

#### You may select as many answers as are appropriate.

Contributor - on the ground monitoring or management; Contributor - scientific research; Advisor (to provide advice or ideas, or to be consulted with)

#### 4. Please state whether you/your organisation holds a particular stance on beaver reintroduction, and whether this has in any way changed over the course of the Trial?

To undertake impartial research to understand the impacts of the return of beaver to great Britain and inform policy.

#### 5. What were the main motivations for you/your organisation to participate in the Trial?

Research

#### 6. Please describe any potential risks or challenges you believe there to have been of you/your organisation's participation in the Trial.

Maintaining scientific integrity and impartiality. Whilst our role as redacted researchers is to undertake independent research, our position as a main project partners has at times led some to question this. Have sought to address via peer review of results etc.

### SECTION 3: Trial Review

This section will question your views on the Trial itself, and what you feel the Trial may or may not have achieved.

#### **7.** Do you consider the Trial structure to have been appropriate for conducting the Trial?

#### Please give reasons for your answer, including any changes you would suggest, if appropriate.

#### ‘Trial structure’ in this question refers to the existence of and relationships between the Licensing Group, Steering Group, Management Strategy Framework Working Group, Science & Evidence Forum, Fisheries Forum and Community Forum. It also refers to the relationship between these groups and the on-the-ground monitoring by Devon Wildlife Trust.

Yes

#### **8.** Do you consider the composition of organisations on the group(s) upon which you sat (SG/BMSF/S&E) to have been appropriate?

#### Please give reasons for your answer, including any changes you would suggest, if appropriate.

Yes

#### **9.** Please detail here if you believe there to have been any key successes of the Trial.

A management plan co-created by a broad spectrum of project partners. A science and evidence report presenting a large body of novel and robust research. High degree of public engagement and support.

#### **10.** Please detail here if you believe there to have been any key failures of the Trial.

#### **11.** Overall, and on balance, have you found your experience of participating in the Trial to have been of value for you/your organisation?

Yes

#### Please use this space to give a reason for your answer to question 11.

Enabled us to undertake a suite of research into a wild living beaver population in a modern intensively managed British landscape.

### SECTION 4: Lessons for the Future

#### **12.** From your experience of this Trial, what do you believe are the key lessons learned for considering potential future (re)introductions of beavers or other species?

That beavers populations can thrive in our landscapes. Results from early stages of beaver catchment colonisation indicate overall environmental impacts are positive. Most conflict or perceived conflict can be managed, but this does require a clear management plan etc.

#### **13.** Following your experience of this Trial, please tell us whether or not you believe potential future (re)introductions of beavers or other species should be subject to a similar Trial process. Please give reasons for your answer.

No. Whilst the trial demonstrates reintroduction of beaver into British landscapes does require management, due to knowledge gained in this trial and others (i.e. work in Scotland and across Europe) expertise and management plans now exist. This should allow future reintroductions to occur with a management plan and where appropriate an associated suite of research opportunities ,but without the extensive (and very expensive) process that has been required for the River Otter (or Knapdale) beaver trials.

#### **14.** Following your experience from this Trial, would you/your organisation consider participating in potential future trial (re)introductions of beavers or other species?

#### Please give reasons for your answer, including what would incentivise your participation.

Yes for two key reasons. 1) Still a range of important and fascinating research to be undertaken 2) Our expertise from this and other beaver projects can help guide future trials.

### Section 5: Final Additional Comments

#### Please use this space to provide any additional comments you may have.

## PARTICIPANT 2 (P2)

### SECTION 2: Your involvement in the Trial

The next three questions will ask you about which group(s) you sat on. For each one that you indicate, you will then be asked to indicate your role upon that group from a check-list.

#### **1.** Were you a member of the ROBT Steering Group (SG)?

Yes

#### Please use the following checklist to indicate your role(s) upon the Steering Group (SG). You may select as many answers as are appropriate.

Advisor (to provide advice or ideas, or to be consulted with); Informer (to inform of wider policy or developments); Informee (to be kept informed of developments)

#### **2.** Were you a member of the ROBT Beaver Management Strategy Framework Working Group (BMSF)?

No

#### Please use the following checklist to indicate your role(s) upon the ROBT Beaver Management Strategy Framework Working Group (BMSF).You may select as many answers as are appropriate.

*This question was not displayed to the respondent*.

#### **3.** Were you a member of the ROBT Science & Evidence Forum?

No

#### Please use the following checklist to indicate your role(s) upon the ROBT Science & Evidence Forum (S&E).

#### You may select as many answers as are appropriate.

*This question was not displayed to the respondent*.

Please state whether you/your organisation holds a particular stance on beaver reintroduction, and whether this has in any way changed over the course of the Trial?

My organisation has expressed conditional support for the project, but does not have a clearly established position on beaver reintroduction.

#### **5.** What were the main motivations for you/your organisation to participate in the Trial?

My organisation has a range of interests / statutory functions that had the potential to be directly affected by the presence of beavers within the catchment (i.e. highways, flood risk management, land-owning interests etc.). On this basis, ever since DWT first considered establishing the Trial, our organisation had highlighted practical issues that we felt needed to be satisfactorily addressed through it through appropriate risk assessment, monitoring, management and mitigation measures.

#### **6.** Please describe any potential risks or challenges you believe there to have been of you/your organisation's participation in the Trial.

The risks identified prior to the commencement of the Trail included: Potential risks to county highway network, with related public safety risks (e.g. trees felled by beavers onto the highway, or indirect effecting such as flooding of highway due to beaver activity); Related risks linked to the public right of way network (i.e. part of the statutory highway), both from a public safety perspective but also given implications for ongoing access in the event of the blocking of paths etc.. Potential flood risks or need for active engagement related to our role as lead local flood authority (plus in our associated

redacted role relating to flood risk). This included the potential for blocking of culverts or other beaver activity potentially exacerbating local flood risk. There was a specific nervousness about this prior to the trial because of the severity of local surface water flooding in settlements in the catchment (e.g. repeated flooding of the school at redacted); Any potential implications for the organisation as a landowner in the catchment, specifically related to the presence of a redacted holding directly alongside the lower reaches of the River Otter. The level of concern relating to these potential risks abated during the trial, reflecting the generally low level of such impacts and the effective approaches to monitoring, management and mitigation deployed by the Project Team. There was an initial risk that our staff may need to devote considerable time to working with the Project Team and affected land owners in dealing with any formal consents required linked our flood risk duties (i.e. for works affecting an Ordinary Watercourse). However, in the event, there was only a very limited need for such input during the Trial.

### SECTION 3: Trial Review

This section will question your views on the Trial itself, and what you feel the Trial may or may not have achieved.

#### **7.** Do you consider the Trial structure to have been appropriate for conducting the Trial?

#### Please give reasons for your answer, including any changes you would suggest, if appropriate.

#### ‘Trial structure’ in this question refers to the existence of and relationships between the Licensing Group, Steering Group, Management Strategy Framework Working Group, Science & Evidence Forum, Fisheries Forum and Community Forum. It also refers to the relationship between these groups and the on-the-ground monitoring by Devon Wildlife Trust.

Yes, a very good Trial structure was established which provided (relatively) clear governance arrangements and excellent opportunities for stakeholder engagement. Two minor observations: 1. The relationship between the Project Team (or Management Group or whatever it was called) and the Steering Group was not made sufficiently clear. This might reflect the fact that this was self-evident to those leading / most closely involved in the Trial; but for others, who had no direct sight of this, the respective roles could have been made a little clearer. 2. There was a degree of overlap and duplication of effort between the Trial structures set up by DWT / the Project Team and the 'Licensing Group' which was established by Natural England. Whilst there was a clear distinction between respective roles, at times the discussion within the Licensing Group may have gone slightly beyond what was necessary from the licensing perspective; there was also some, perhaps unavoidable, duplication of reporting for those involved in both.

#### **8.** Do you consider the composition of organisations on the group(s) upon which you sat (SG/BMSF/S&E) to have been appropriate?

#### Please give reasons for your answer, including any changes you would suggest, if appropriate.

Yes. Although quite large, the Steering Group did appear to provide opportunity for representation by key stakeholders (i.e. those directly involved in the Trial or with the potential to be significantly affected by it, plus relevant statutory interests). However, one important point is that the successful operation of the Steering Group was the willingness of the individual participants to engage positively (i.e. highlighting relevant concerns as necessary, but in a manner which sought to resolve these in a mutually acceptable manner). There were tensions between various stakeholders involved in or affected by the Trial, but the Steering Group helped to manage these well.

#### **9.** Please detail here if you believe there to have been any key successes of the Trial.

I consider the Trial to be one of the most successful conservation initiatives that I have witnessed or been involved in during over 30 years ofemployment in the environmental sector in Devon. Key points relevant to this success include: The very strong lead provided by DWT and their unstinting commitment to the project; The competency, professionalism, commitment and dedication of the entire project team; The extremely strong focus on community and stakeholder engagement, which was instrumental in getting support from such a wide range of individuals and organisations; Strong governance arrangements and structures, as outlined above, which provided clear accountability and good opportunities for active participation / input by relevant stakeholders; The strong support provided by the redacted as the key landowner (i.e. it provide to be extremely fortunate for the project for there to be such a dominant landowner, but it did make it absolutely vital to get them fully onboard and engaged from the start); The strength of the research / monitoring programme and those undertaking this work, with this forming an absolutely integral and appropriately funded element of the project throughout; The establishment and maintenance of a strong mitigation strategy, to deal with issues quickly and effectively as they arose, as without this there would have been many more people opposed to the presence of the beavers; Finally, the successful colonisation of the catchment by beavers and the apparent health of the population, which was definitely supported by the active intervention of the project (esp. through the approach to releases and through the veterinary checks and supervision).

#### **10.** Please detail here if you believe there to have been any key failures of the Trial.

Although wrong to characterise it as a failure, it is a shame that there couldn't have been a more definitive conclusion on the impact of beavers on migratory fish populations, which appears to remain as one of the points of contention. Although the Trial provided some good evidence on this issue, the work wasn't sufficiently comprehensive or of sufficient duration to enable a clear conclusion and consensus to be achieved. One further point, again which cannot be expressed as a failure, but was a possible limitation of the project, was (ironically) the lack of more problems. Although the beavers did create 'issues' in a number of locations which required active management to mitigate the potential consequences, which proved to be extremely instructive element of the Trial, it might have been better if there had been even more of these types of localised problem. In particular, I'm not convinced that the Trial has, properly or adequately, addressed potential risks to public safety linked to the widespread presence of beavers. Having said that, it is unrealistic to expect too much of a single Trial!

#### **11.** Overall, and on balance, have you found your experience of participating in the Trial to have been of value for you/your organisation?

Yes

#### Please use this space to give a reason for your answer to question 11.

Firstly, it has helped to demonstrate that the initially perceived risks (from the presence / activities of beavers) were not nearly as severe in practice as some feared they might be. Secondly, the Trial has demonstrated how effectively conflicts and issues can be appropriately managed through a clear and sufficiently resourced strategy and dedicated team. Both of these points help to build strength in the case for allowing the further introduction and spread of beavers, PROVIDED that this is matched by the establishment of a suitable management framework.

### SECTION 4: Lessons for the Future

#### **12.** From your experience of this Trial, what do you believe are the key lessons learned for considering potential future (re)introductions of beavers or other species?

The key lessons are that: beavers are not like most other species which are subject to reintroduction programmes; there is strong evidence of the benefits which derive from the presence of beavers in a catchment (environmental and, potentially, economic); but the realisation of these benefits, whilst adequately managing the localised difficulties and problems which can arise does require the establishment of a clear and suitably resourced management / mitigation framework; such introductions are most successful if supported by a much wider approach which informs, support and engages all those potentially affected by it.

#### **13.** Following your experience of this Trial, please tell us whether or not you believe potential future (re)introductions of beavers or other species should be subject to a similar Trial process. Please give reasons for your answer.

I am not convinced that future releases do require a similar Trial process. However, it does require a clearly managed and resourced process to support either the natural spread and colonisation of beavers or, perhaps, a planned introduction programme. If that proves not be practical, then a revised and refined Trial approach might form the next step, in order to broaden the experience, plug gaps in understanding and to establish greater clarity on how the species might, ultimately, expand, flourish and be subject to appropriate ongoing management as a native species.

#### **14.** Following your experience from this Trial, would you/your organisation consider participating in potential future trial (re)introductions of beavers or other species?

Please give reasons for your answer, including what would incentivise your participation.

My organisation might need to have some ongoing involvement in the management of beavers, either through future trials or through more widespread approaches adopted across the county, just to ensure that relevant issues / risks relating to our statutory functions are appropriately handled.

### Section 5: Final Additional Comments

#### Please use this space to provide any additional comments you may have.

None...I've said too much already!

## PARTICIPANT 3 (P3)

### SECTION 2: Your involvement in the Trial

The next three questions will ask you about which group(s) you sat on. For each one that you indicate, you will then be asked to indicate your role upon that group from a check-list.

#### **1.** Were you a member of the ROBT Steering Group (SG)?

Yes

#### Please use the following checklist to indicate your role(s) upon the Steering Group (SG). You may select as many answers as are appropriate.

Advisor (to provide advice or ideas, or to be consulted with); Informer (to inform of wider policy or developments)

#### **2.** Were you a member of the ROBT Beaver Management Strategy Framework Working Group (BMSF)?

No

#### Please use the following checklist to indicate your role(s) upon the ROBT Beaver Management Strategy Framework Working Group (BMSF).You may select as many answers as are appropriate.

*This question was not displayed to the respondent*.

#### **3.** Were you a member of the ROBT Science & Evidence Forum?

No

#### Please use the following checklist to indicate your role(s) upon the ROBT Science & Evidence Forum (S&E).

#### You may select as many answers as are appropriate.

*This question was not displayed to the respondent*.

#### **4.** Please state whether you/your organisation holds a particular stance on beaver reintroduction, and whether this has in any way changed over the course of the Trial?

Redacted were initially uncertain about beaver reintroduction (some years before River Otter beavers became public knowledge). As a result of considerable advocacy work by both in-house ecologists, and independent advisers (notably redacted) we took up a stronger positive outlook and adopted and initiated two beaver "reintroductions" (both within enclosed pens)...in the redacted and later in redacted. These were enthusiastically supported by most of staff (though not all as seen by some not so much as a threat to forests but a distraction to core forestry management and timber production activity). We baulked at an "open" release project in redacted as a result of inherent caution, but are now close to a valley wide reintroduction in redacted. Redacted now I think a strong supporter and advocate, provided done with all regard to legal and scientific protocols adhered to, and perhaps, as importantly, seen as acceptable to neighbouring landowners and their interests. Participaton in ROBT has expanded support within redacted and crucially allowed the Rangerteam to both learn from project ad to contribute to practicalities of future management.

#### **5.** What were the main motivations for you/your organisation to participate in the Trial?

I was very keen to ensure two things: One was that the practical experience of lethal control and humane trapping (and deporting or humane despatch) of wildlife from our Wildlife Ranger team, acquired through the use of firearms, the management of deer and trapping of mink across the country, was made available to the ROBT for development of the "post release"management of beavers once they become abundant across the UK landscape. ... and secondly I was very keen for redacted to both be involved and to be seen internally to be involved, thus promoting both the role of the rangers in a wider conservation programme of considerable import and excitement, and to bring the knowledge and experience of the ROBT to bear on redacted landscapes. Our involvement also of course legitimised the recovery of beavers as an aim within the redacted land management .

#### **6.** Please describe any potential risks or challenges you believe there to have been of you/your organisation's participation in the Trial.

None in hindsight... though we were I think seen as rather too keen on lethal control by others! This is a slightly misleading interpretation... we are I think keen to see beavers as widespread and as common as deer and foxes, but to reach that goal more strong acceptance amongst more hostile land owners would be required. By introducing the subject of means of humane and safe lethal control, as used and advocated by professionals in redacted (nearly all of whom are keen naturalists) from the outset we hoped to allay fears amongst others (farmers and foresters alike) that should they have problems with beavers then a range of options for management (including lethal control) had been thought through. I think we achieved that. We are many years away from beavers becoming common enough for the full range of control measures to be needed or applied, but we get there in decades not centuries! So no real risk though some (unfounded) reputational risks re our ready use of firearms perhaps were borne; more widely the public has expressed huge support for our role in introducing beavers to the public in the redacted and near redacted. There has been some rolling of eyes amongst the forest industry, but no real damage to redacted reputation. And this of course resulting from our direct engagement with beavers, not via our engagement with ROBT.

### SECTION 3: Trial Review

This section will question your views on the Trial itself, and what you feel the Trial may or may not have achieved.

#### **7.** Do you consider the Trial structure to have been appropriate for conducting the Trial?

#### Please give reasons for your answer, including any changes you would suggest, if appropriate.

#### ‘Trial structure’ in this question refers to the existence of and relationships between the Licensing Group, Steering Group, Management Strategy Framework Working Group, Science & Evidence Forum, Fisheries Forum and Community Forum. It also refers to the relationship between these groups and the on-the-ground monitoring by Devon Wildlife Trust.

Am not sure I have a lot to add here..... beyond detail it does look, and certainly felt a little over complex and complicated. One could argue it was very expensive but probably has to be seen in terms of recovery of beavers across UK, or certainly lowland England, and not as a cost simply to recovery River Otter, or Devon and South west. Not a model replicable i all such small catchments ...perhaps applicable to Thames or Severn catchments etc? I am uncertain but its structure was in part determined i think by redacted insistence on a complex (and very expensive!) trial to allow them to escape any blame for allowing beavers to be retained in landscape? The costs were largely passed on to others notably the Devon Wildlife Trust. So.... framework could be simpler almost certainly. The science and monitoring activity on the ground in Devon was exemplary. and worth noting that the studies in Boldventure complemented the Otter work really well...that too could be a model elsewhere where enclosed trials in similar situations might support restoration in wider landscapes with similar features or vegetation.

#### **8.** Do you consider the composition of organisations on the group(s) upon which you sat (SG/BMSF/S&E) to have been appropriate?

#### Please give reasons for your answer, including any changes you would suggest, if appropriate.

It appeared to us (I attended with redacted) that all interests were represented and that the individuals were well suited to the task. I was aware that many seemed personally fascinated and supportive of having beavers in landscape and occasionally had to say things that reported on or represented their members views or concerns, rather than their own more informed take on things. so a good composition, backed by some excellent individual reps. Worked well..... could be really challenging elsewhere!

#### **9.** Please detail here if you believe there to have been any key successes of the Trial.

Dissemination of much deeper understanding throughout group...from beaver ecology to farmers and fishermen for example, but also the reverse,from fishing concerns and preoccupations and local economic interests and constraints upwards to beaver enthusiasts and ecologists. All such groups benefit from this flow and counterflow of understanding and it was very well done with ROBT 2. their ability to get excellent science (notably from Exeter University) and experience from across Europe and North America to members of group that may not have had time or access in any other way. 3. The role of redacted in providing logistical, mtg space and practical support was excellent... a key to success, but also a success in its own right. The redacted is very influential amongst so many forward thinking large estates across the Country. Securing their overt and cheerful support was a great success..and a tribute to Devon WT reputation developed over the years running up to the beavers arrival in Devon.

#### **10.** Please detail here if you believe there to have been any key failures of the Trial.

Its far too costly and complex an approach to be adopted or advocated in other catchments...it needs to be seen as a proxy for other catchments andnot a model. 2. cannot think of a no.2!

11. Overall, and on balance, have you found your experience of participating in the Trial to have been of value for you/your organisation?

Yes

#### Please use this space to give a reason for your answer to question 11.

It has helped place redacted as a key partner in reintroduction and rewilding programmes. It has provided us with considerable new insights and access to information on the role of beavers and their management. It provided us with access to redacted staff and key locations to arrange staff visits and training sessions of direct relevance to work elsewhere.

### SECTION 4: Lessons for the Future

#### **12.** From your experience of this Trial, what do you believe are the key lessons learned for considering potential future (re)introductions of beavers or other species?

Science and monitoring needs to well planned and well executed. Public, institutional and private understanding of the true nature of beavers, and the impact they may have (both beneficial and negative), needs to be both professional and accurate, and widely disseminated to a wide range of stakeholders and interested parties. Professional "beaver officers" are likely to be a key to successful integration of beavers into wider catchments.

#### **13.** Following your experience of this Trial, please tell us whether or not you believe potential future (re)introductions of beavers or other species should be subject to a similar Trial process. Please give reasons for your answer.

No.... I believe that the beaver can and will spread far more widely, and indeed should be encouraged to do so. But the level of expense and input in this trial paves the way for understanding and insight.... it is not as a model for more catchments. It provided the lessons, not the model. So for a large catchment or even a similar one in scale (say the Itchen/Test here in Hampshire) the lessons should be drawn from the Otter, and a small team of staff employed, but the level of mapping and science, monitoring and discussion cannot and should not be replicated. New science perhaps, but not repeated science. Beaver officers are minimum requirement I believe, but much of rest dos not need to be copied in each and every situation unless it adds to our understanding in new and untested situations...which the Test as a fly river may well do... but not as a matter of rote.

#### **14.** Following your experience from this Trial, would you/your organisation consider participating in potential future trial (re)introductions of beavers or other species?

#### Please give reasons for your answer, including what would incentivise your participation.

Having retired from redacted this is not in my gift to answer but I would be surprised if redacted declined. A lot might depend on the nature of the catchment, the extent of forestry activity in the area, or redacted landholding within it and the role of the redacted estate...as a location of lodes and dams for example. The new redacted Senior Ecologist, redacted, would be delighted! He worked on beavers in Scotland before joining us in the Dean.

### Section 5: Final Additional Comments

#### Please use this space to provide any additional comments you may have.

It was a rare privilege to have been involved with the ROBT. And I cannot commend the DWT nor the Clinton Devon Estate enough. The approach was imaginative, committed, pragmatic and cheerful throughout. I look forward to hearing more of the Otter Beavers in coming years... and hopefully might get to see one one day!

## PARTICIPANT 4 (P4)

### SECTION 2: Your involvement in the Trial

The next three questions will ask you about which group(s) you sat on. For each one that you indicate, you will then be asked to indicate your role upon that group from a check-list.

#### **1.** Were you a member of the ROBT Steering Group (SG)?

Yes

#### Please use the following checklist to indicate your role(s) upon the Steering Group (SG). You may select as many answers as are appropriate.

Contributor - on the ground monitoring or management; Contributor - scientific research; Advisor (to provide advice or ideas, or to be consulted with); Informer (to inform of wider policy or developments); Informee (to be kept informed of developments); Reporter (lead for group output reports)

#### **2.** Were you a member of the ROBT Beaver Management Strategy Framework Working Group (BMSF)?

Yes

#### Please use the following checklist to indicate your role(s) upon the ROBT Beaver Management Strategy Framework Working Group (BMSF).You may select as many answers as are appropriate.

Contributor - on the ground monitoring or management; Contributor - scientific research; Advisor (to provide advice or ideas, or to be consulted with); Informer (to inform of wider policy or developments); Informee (to be kept informed of developments); Reporter (lead for group output reports)

#### **3.** Were you a member of the ROBT Science & Evidence Forum?

Yes

#### Please use the following checklist to indicate your role(s) upon the ROBT Science & Evidence Forum (S&E).

#### You may select as many answers as are appropriate.

Chair or Convenor; Contributor - on the ground monitoring or management; Contributor - scientific research Advisor (to provide advice or ideas, or to be consulted with); Informer (to inform of wider policy or developments); Informee (to be kept informed of developments); Reporter (lead for group output reports)

#### **4.** Please state whether you/your organisation holds a particular stance on beaver reintroduction, and whether this has in any way changed over the course of the Trial?

Redacted is promoting the reintroduction of beavers back into Britain.

#### **5.** What were the main motivations for you/your organisation to participate in the Trial?

Hugh range of benefits in terms of ecosystems, natural processes, ecosystem services. Beavers are a keystone species, without which our ecosystems are in poorer condition.

6. Please describe any potential risks or challenges you believe there to have been of you/your organisation's participation in the Trial.

Beaver reintroduction is controversial in some quarters (esp farmers and anglers) and so adopting such a positive stance risks alientating some stakeholder groups - such as the farmers that we work with. There have also been financial risks associated with the Trial - in particular the resources that had to be put aside for implementing the Exit Strategy, and for compensating for any significant impacts - which may need to have been covered by our insurance (eg Flooding of properties that we could have been responsible for under the licence).

### SECTION 3: Trial Review

This section will question your views on the Trial itself, and what you feel the Trial may or may not have achieved.

#### **7.** Do you consider the Trial structure to have been appropriate for conducting the Trial?

#### Please give reasons for your answer, including any changes you would suggest, if appropriate.

#### ‘Trial structure’ in this question refers to the existence of and relationships between the Licensing Group, Steering Group, Management Strategy Framework Working Group, Science & Evidence Forum, Fisheries Forum and Community Forum. It also refers to the relationship between these groups and the on-the-ground monitoring by Devon Wildlife Trust.

This structure mostly seemed to work well. It felt overly complicated at times, but this allowed lots of different stakehodlers to be involved and feed their thoughts and feelings into the process.

#### **8.** Do you consider the composition of organisations on the group(s) upon which you sat (SG/BMSF/S&E) to have been appropriate?

#### Please give reasons for your answer, including any changes you would suggest, if appropriate.

Mostly - there were a few organisations and individuals that didn't participate - despite being invited and wanting to be involved. Due to my time pressures and for other reasons, the forums varied significantly in terms of how often they got together and what they achieved.

#### **9.** Please detail here if you believe there to have been any key successes of the Trial.

Whether the Trial has been a success in terms of reintroducing beavers into England remains to be seen....but it feels like we've done the best we could in terms of attracting community support, and gathering the evidence together to allow the government to make a decision on their future.

#### **10.** Please detail here if you believe there to have been any key failures of the Trial.

There are some stakeholder groups that have not been as successfully engaged with - eg the Angling groups.

11. Overall, and on balance, have you found your experience of participating in the Trial to have been of value for you/your organisation?

Yes

#### Please use this space to give a reason for your answer to question 11.

### SECTION 4: Lessons for the Future

#### **12.** From your experience of this Trial, what do you believe are the key lessons learned for considering potential future (re)introductions of beavers or other species?

Doing lots of outreach work has been vitally important - and being open and honest about the conflicts that beavers can cause has helped with lots of stakeholder groups. Our presentations are balanced rather than overly positive, which I think has helped. Bringing lots of stakeholders together, and providing them with lots of feedback and reports hopefully helped too - they were a lot of work, so lets hope they were useful. Running a successful project requires huge focus and dedication far and above the normal 9-5 working practices.

#### **13.** Following your experience of this Trial, please tell us whether or not you believe potential future (re)introductions of beavers or other species should be subject to a similar Trial process. Please give reasons for your answer.

Other species, yes, but other beaver projects should not require the same level of research and justification. The evidence is now there to make the decision about this native species, and once it is recognised as resident again, it needs to reintroduced nationally.

#### **14.** Following your experience from this Trial, would you/your organisation consider participating in potential future trial (re)introductions of beavers or other species?

#### Please give reasons for your answer, including what would incentivise your participation.

Yes

### Section 5: Final Additional Comments

#### Please use this space to provide any additional comments you may have.

## PARTICIPANT 5 (P5)

### SECTION 2: Your involvement in the Trial

The next three questions will ask you about which group(s) you sat on. For each one that you indicate, you will then be asked to indicate your role upon that group from a check-list.

#### **1.** Were you a member of the ROBT Steering Group (SG)?

No

#### Please use the following checklist to indicate your role(s) upon the Steering Group (SG). You may select as many answers as are appropriate.

*This question was not displayed to the respondent*.

#### **2.** Were you a member of the ROBT Beaver Management Strategy Framework Working Group (BMSF)?

Yes

#### Please use the following checklist to indicate your role(s) upon the ROBT Beaver Management Strategy Framework Working Group (BMSF).You may select as many answers as are appropriate.

Advisor (to provide advice or ideas, or to be consulted with)

#### **3.** Were you a member of the ROBT Science & Evidence Forum?

No

#### Please use the following checklist to indicate your role(s) upon the ROBT Science & Evidence Forum (S&E).

#### You may select as many answers as are appropriate.

*This question was not displayed to the respondent.*

#### **4.** Please state whether you/your organisation holds a particular stance on beaver reintroduction, and whether this has in any way changed over the course of the Trial?

None

#### **5.** What were the main motivations for you/your organisation to participate in the Trial?

Represent the farming community and learn. Thus providing more rounded info back to the farming community.

#### **6.** Please describe any potential risks or challenges you believe there to have been of you/your organisation's participation in the Trial.

None

### SECTION 3: Trial Review

This section will question your views on the Trial itself, and what you feel the Trial may or may not have achieved.

#### **7.** Do you consider the Trial structure to have been appropriate for conducting the Trial?

#### Please give reasons for your answer, including any changes you would suggest, if appropriate.

#### ‘Trial structure’ in this question refers to the existence of and relationships between the Licensing Group, Steering Group, Management Strategy Framework Working Group, Science & Evidence Forum, Fisheries Forum and Community Forum. It also refers to the relationship between these groups and the on-the-ground monitoring by Devon Wildlife Trust.

Yes, but the dubious nature of how the beavers arrived is always a bone of contention amonst farmers.

#### **8.** Do you consider the composition of organisations on the group(s) upon which you sat (SG/BMSF/S&E) to have been appropriate?

#### Please give reasons for your answer, including any changes you would suggest, if appropriate.

Yes, though some actual farmers not the likes of me or redacted would help.

#### **9.** Please detail here if you believe there to have been any key successes of the Trial.

knowledge exchange.

#### **10.** Please detail here if you believe there to have been any key failures of the Trial.

no key failures.

#### **11.** Overall, and on balance, have you found your experience of participating in the Trial to have been of value for you/your organisation?

Yes

#### Please use this space to give a reason for your answer to question 11.

Better placed to field questions from local farmers about the impacts and controls should beavers make their way to their land.

### SECTION 4: Lessons for the Future

#### **12.** From your experience of this Trial, what do you believe are the key lessons learned for considering potential future (re)introductions of beavers or other species?

Whatever happens, structured control will be necessary, otherwise there could be some disasters in adhoc control taking place.

#### **13.** Following your experience of this Trial, please tell us whether or not you believe potential future (re)introductions of beavers or other species should be subject to a similar Trial process. Please give reasons for your answer.

This trial should help inform people so that any reintroductions should be carried out in theist places with limited initial impact. This would allow time for the local communities to get used to the idea of having them back.

#### **14.** Following your experience from this Trial, would you/your organisation consider participating in potential future trial (re)introductions of beavers or other species?

#### Please give reasons for your answer, including what would incentivise your participation.

Yes

### Section 5: Final Additional Comments

#### Please use this space to provide any additional comments you may have.

None.

## PARTICIPANT 6 (P6)

### SECTION 2: Your involvement in the Trial

The next three questions will ask you about which group(s) you sat on. For each one that you indicate, you will then be asked to indicate your role upon that group from a check-list.

#### **1.** Were you a member of the ROBT Steering Group (SG)?

Yes

#### Please use the following checklist to indicate your role(s) upon the Steering Group (SG). You may select as many answers as are appropriate.

Chair or Convenor; Contributor - on the ground monitoring or management; Contributor - scientific research; Advisor (to provide advice or ideas, or to be consulted with); Informer (to inform of wider policy or developments); Reporter (lead for group output reports); Funder (contributing funder for the ROBT)

#### **2.** Were you a member of the ROBT Beaver Management Strategy Framework Working Group (BMSF)?

Yes

Please use the following checklist to indicate your role(s) upon the ROBT Beaver Management Strategy Framework Working Group (BMSF).You may select as many answers as are appropriate.

Chair or Convenor; Contributor - on the ground monitoring or management; Contributor - scientific research; Advisor (to provide advice or ideas, or to be consulted with); Informer (to inform of wider policy or developments); Reporter (lead for group output reports); Funder (contributing funder for the ROBT)

#### **3.** Were you a member of the ROBT Science & Evidence Forum?

Yes

#### Please use the following checklist to indicate your role(s) upon the ROBT Science & Evidence Forum (S&E).

#### You may select as many answers as are appropriate.

Contributor - on the ground monitoring or management; Contributor - scientific research; Advisor (to provide advice or ideas, or to be consulted with); Informer (to inform of wider policy or developments); Informee (to be kept informed of developments); Funder (contributing funder for the ROBT)

#### **4.** Please state whether you/your organisation holds a particular stance on beaver reintroduction, and whether this has in any way changed over the course of the Trial?

We wish to see an ambitious strategy for beaver reintroduction throughout Devon and the UK.

#### **5.** What were the main motivations for you/your organisation to participate in the Trial?

Redacted. Recognition of the keystone role beavers will play in the future health of our ecosystems.

#### **6.** Please describe any potential risks or challenges you believe there to have been of you/your organisation's participation in the Trial.

Reputational - divisive issues - potential failure or unsustainable impacts from beavers Financial - we underwrote the entire trial Social - partnership interactions and potential breakdown Success - potential that the trial was not a success and beavers not permitted to remain

### SECTION 3: Trial Review

This section will question your views on the Trial itself, and what you feel the Trial may or may not have achieved.

#### **7.** Do you consider the Trial structure to have been appropriate for conducting the Trial?

#### Please give reasons for your answer, including any changes you would suggest, if appropriate.

#### ‘Trial structure’ in this question refers to the existence of and relationships between the Licensing Group, Steering Group, Management Strategy Framework Working Group, Science & Evidence Forum, Fisheries Forum and Community Forum. It also refers to the relationship between these groups and the on-the-ground monitoring by Devon Wildlife Trust.

Very good. However full engagement and support from members was essential and not forthcoming from certain quarters.

#### **8.** Do you consider the composition of organisations on the group(s) upon which you sat (SG/BMSF/S&E) to have been appropriate?

#### Please give reasons for your answer, including any changes you would suggest, if appropriate.

Yes. However input was not always representative of the organisation's members.

#### **9.** Please detail here if you believe there to have been any key successes of the Trial.

It was a success! SG members engaged well and we maintained an open and transparent dialogue. Science and evidence work was first class. Community engagement enabling people to learn to live alongside the animals once again. Mitigation and future management agreed. BMSF All reporting All partners acting in the interests of the Trial

#### **10.** Please detail here if you believe there to have been any key failures of the Trial.

Not embedding a national strategic approach. Not resolving deep seated beliefs regarding beaver and fish interactions

#### **11.** Overall, and on balance, have you found your experience of participating in the Trial to have been of value for you/your organisation?

Yes

#### Please use this space to give a reason for your answer to question 11.

### SECTION 4: Lessons for the Future

#### **12.** From your experience of this Trial, what do you believe are the key lessons learned for considering potential future (re)introductions of beavers or other species?

Follow a similar approach - there are few trials which end where all partners / members agree on success. Open, inclusive and transparent approach. Hold the middle ground. Tease out key issues from the outset that are likely to fester. Remember - these are social science trials as well as species reintroductions!

#### **13.** Following your experience of this Trial, please tell us whether or not you believe potential future (re)introductions of beavers or other species should be subject to a similar Trial process. Please give reasons for your answer.

Yes, however the risk with anything new is that management systems and processes are overly complex and intensive. We are facing a climate and ecological crisis - species reintroductions need to be done well - but the barriers organisations face are often prohibitive and costly.

#### **14.** Following your experience from this Trial, would you/your organisation consider participating in potential future trial (re)introductions of beavers or other species?

#### Please give reasons for your answer, including what would incentivise your participation.

Yes - a more support legislative framework and recognition we are helping to solve major problems. This needs to be an 'in it together' approach rather than one organisation shouldering all the risk.

### Section 5: Final Additional Comments

#### Please use this space to provide any additional comments you may have.

## PARTICIPANT 7 (P7)

### SECTION 2: Your involvement in the Trial

The next three questions will ask you about which group(s) you sat on. For each one that you indicate, you will then be asked to indicate your role upon that group from a check-list.

#### **1.** Were you a member of the ROBT Steering Group (SG)?

Yes

#### Please use the following checklist to indicate your role(s) upon the Steering Group (SG). You may select as many answers as are appropriate.

Advisor (to provide advice or ideas, or to be consulted with); Informer (to inform of wider policy or developments); Informee (to be kept informed of developments)

#### **2.** Were you a member of the ROBT Beaver Management Strategy Framework Working Group (BMSF)?

No

#### Please use the following checklist to indicate your role(s) upon the ROBT Beaver Management Strategy Framework Working Group (BMSF).You may select as many answers as are appropriate.

*This question was not displayed to the respondent*.

#### **3.** Were you a member of the ROBT Science & Evidence Forum?

No

#### Please use the following checklist to indicate your role(s) upon the ROBT Science & Evidence Forum (S&E).

#### You may select as many answers as are appropriate.

*This question was not displayed to the respondent.*

#### **4.** Please state whether you/your organisation holds a particular stance on beaver reintroduction, and whether this has in any way changed over the course of the Trial?

In the broadest sense, we are aware that there are opportunities and risks associated with the return of beavers to rivers in England. We have been working and continue to work with other organisations and projects such as the River Otter Beaver Trial, to better understand these opportunities and risks, help us inform and advise defra on their future policy on beavers, and be best prepared to deliver and support any management measures that may be required by us and others. This Project has definitely given us more evidence, and insight into these opportunities and risks, and so we are already in a stronger position to do these things.

#### **5.** What were the main motivations for you/your organisation to participate in the Trial?

To support and learn from a project that has been all about trying to better understand the opportunities and risks associated with beavers in the wild in England.

#### **6.** Please describe any potential risks or challenges you believe there to have been of you/your organisation's participation in the Trial.

1) Concern that some might see our association with the Steering Group and the Project as us having already made up our mind on beavers returning to the wild. 2) Resource demands and commitments 3) the prospect of being associated with what might be seen as very challenging and controversial decisions, at any stage during the project and, at the end, depending on government decision on the future of the beavers in the wild.

### SECTION 3: Trial Review

This section will question your views on the Trial itself, and what you feel the Trial may or may not have achieved.

#### **7.** Do you consider the Trial structure to have been appropriate for conducting the Trial?

#### Please give reasons for your answer, including any changes you would suggest, if appropriate.

#### ‘Trial structure’ in this question refers to the existence of and relationships between the Licensing Group, Steering Group, Management Strategy Framework Working Group, Science & Evidence Forum, Fisheries Forum and Community Forum. It also refers to the relationship between these groups and the on-the-ground monitoring by Devon Wildlife Trust.

In terms of structure, I feel it has worked very well. From where I have been, I have felt that the focus, engagement and chain of command across these groups seems to have worked well. Even though we have been more involved in some of the groups, and less involved in others, I and my organisation have felt very aware and engaged through the project team, meetings, communications and reports.

#### **8.** Do you consider the composition of organisations on the group(s) upon which you sat (SG/BMSF/S&E) to have been appropriate?

#### Please give reasons for your answer, including any changes you would suggest, if appropriate.

Yes. I believe there has been a good range of interests and organisations given the opportunity to be part of this project, covering landowner and business interests, environmental interests, social and community interests and, within that, a good suite of public, private, charitable and scientific bodies. It has felt well balanced.

#### **9.** Please detail here if you believe there to have been any key successes of the Trial.

Science and Evidence outputs and, including specific reports Excellent communications and engagement leading to positive support for the project and increased awareness and understanding of the opportunities and risks Management of the project to time, with key outputs and key success criteria met

#### **10.** Please detail here if you believe there to have been any key failures of the Trial.

would not say it is a failure, but I believe there remain still, some very strong views and concerns, particularly from fisheries interests about the risks tofish passage and fish habitats from beaver activity. The project has contributed important information to this debate. However, there will only be so far that this could have been taken forward, in what has been a relatively short period, in the context of what beaver activity there has been, and dependent on other factors during this project. I sense this is an area for further work and dialogue, as we go forward.

#### **11.** Overall, and on balance, have you found your experience of participating in the Trial to have been of value for you/your organisation?

Yes

#### Please use this space to give a reason for your answer to question 11.

I and my organisation have learnt massively about beavers in the wild, the opportunities and risks, and management interventions that may be needed insome cases. If it is decided that beavers will stay in the wild, in England, we are better informed and prepared to adapt our own strategies and operations, and advise others in future catchment management approaches, that might involve beavers.

### SECTION 4: Lessons for the Future

#### **12.** From your experience of this Trial, what do you believe are the key lessons learned for considering potential future (re)introductions of beavers or other species?

The importance of a management framework, and local leadership - whether that be a local organisation or local partnership - to bring interested groups and issues `to the table', move things forward and act as a point of contact, particularly in the early phases of any (re)introduction.

#### **13.** Following your experience of this Trial, please tell us whether or not you believe potential future (re)introductions of beavers or other species should be subject to a similar Trial process. Please give reasons for your answer.

As an approach, this has been a very well run and collaborative effort, securing leadership, partnership working and progress on the key objectives of the project. Government decisions clearly still awaited, but as a model, it seems to have worked, and no reason to believe it would not work elsewhere. My thinking is that, although there is always room and need for more science and learning, any such project in the future, here or anywhere else, has to be more orientated around management advice and interventions (where necessary) to help ensure as smooth a transition from beavers being seen as new to the landscape, to the wild, to the way rivers work, to a position where beavers are seen as being a natural part of all that.

#### **14.** Following your experience from this Trial, would you/your organisation consider participating in potential future trial (re)introductions of beavers or other species?

#### Please give reasons for your answer, including what would incentivise your participation.

Again, depending on the government decision. Yes, for continued learning and understanding about beavers, in the wild, about how we adapt our strategies and operations and work with others in catchments, where beavers exist.

### Section 5: Final Additional Comments

#### Please use this space to provide any additional comments you may have.

I just want to commend and thank Devon Wildlife Trust, including the Steering Group Chair - redacted - the project team - particularly Pete, Mark and Jake - andthe Project partners - particularly redacted at Exeter University - for what they have all done to run and engage us and others in what has been a very professional, interesting, and informative piece of very applied learning. Thank you.

## PARTICIPANT 8 (P8)

### SECTION 2: Your involvement in the Trial

The next three questions will ask you about which group(s) you sat on. For each one that you indicate, you will then be asked to indicate your role upon that group from a check-list.

#### **1.** Were you a member of the ROBT Steering Group (SG)?

Yes

#### Please use the following checklist to indicate your role(s) upon the Steering Group (SG). You may select as many answers as are appropriate.

Contributor - scientific research; Advisor (to provide advice or ideas, or to be consulted with); Informer (to inform of wider policy or developments); Informee (to be kept informed of developments)

#### **2.** Were you a member of the ROBT Beaver Management Strategy Framework Working Group (BMSF)?

Yes

#### Please use the following checklist to indicate your role(s) upon the ROBT Beaver Management Strategy Framework Working Group (BMSF).You may select as many answers as are appropriate.

Contributor - scientific research; Advisor (to provide advice or ideas, or to be consulted with); Informer (to inform of wider policy or developments); Informee (to be kept informed of developments)

#### **3.** Were you a member of the ROBT Science & Evidence Forum?

Yes

#### Please use the following checklist to indicate your role(s) upon the ROBT Science & Evidence Forum (S&E).

#### You may select as many answers as are appropriate.

Contributor - scientific research; Advisor (to provide advice or ideas, or to be consulted with); Informer (to inform of wider policy or developments); Informee (to be kept informed of developments)

#### **4.** Please state whether you/your organisation holds a particular stance on beaver reintroduction, and whether this has in any way changed over the course of the Trial?

We have a "yes if" approach with an informal approach document explaining the redacted roles and remit. This stance has remained the same and the trial has helped inform further. Wouldn't say it had substantially changed.

#### **5.** What were the main motivations for you/your organisation to participate in the Trial?

**Redacted** have a significant role in the water environment operationally, advisory and regulatory. Presence of beavers can influence all of these in varying aspects of our work and also potentially deliver a number of objectives in a positive way, in particular around working with natural processes. There are also aspects where presence may bring challenges and so the trial provides/provided was/is useful and informative.

#### **6.** Please describe any potential risks or challenges you believe there to have been of you/your organisation's participation in the Trial.

**Redacted** works with a wide range of partners and stakeholders who hold various positions regarding beaver reintroduction. Redacted is a large evidence based organisation and internally there are also many views hence the adoption of a cross functional approach document. It was occasionally challenging to work through the aspects of the project in order to provide a view on an area based trial whilst also operating as a national organisation.

### SECTION 3: Trial Review

This section will question your views on the Trial itself, and what you feel the Trial may or may not have achieved.

#### **7.** Do you consider the Trial structure to have been appropriate for conducting the Trial?

#### Please give reasons for your answer, including any changes you would suggest, if appropriate.

#### ‘Trial structure’ in this question refers to the existence of and relationships between the Licensing Group, Steering Group, Management Strategy Framework Working Group, Science & Evidence Forum, Fisheries Forum and Community Forum. It also refers to the relationship between these groups and the on-the-ground monitoring by Devon Wildlife Trust.

The structure seemed sound and effective, Groups I was on were well run, effective and professional with opportunities for all to input. There could have been benefit in more regular meetings of the fisheries forum at earlier stages and links between that group and the Science and evidence forum were not that clear. Redacted had an MoU which was also useful but not aware if there were other arrangements with other groups like this.

#### **8.** Do you consider the composition of organisations on the group(s) upon which you sat (SG/BMSF/S&E) to have been appropriate?

Please give reasons for your answer, including any changes you would suggest, if appropriate.

Overall Yes. Science and evidence group would have benefitted from more redacted input. I understand they were invited but not able to attend.

#### **9.** Please detail here if you believe there to have been any key successes of the Trial.

Strong and effective leadership and running of meetings inviting full participation of members on every occasion. Uni of Exeter involvement in providing academic input and expertise. Public awareness. Pragmatic approach.

#### **10.** Please detail here if you believe there to have been any key failures of the Trial.

No key ones really. Sometimes felt like lessons and experience/expertise from Scotland were not being fully taken into account or utilised and potentially therefore re-inventing the wheel when this was not needed.

#### **11.** Overall, and on balance, have you found your experience of participating in the Trial to have been of value for you/your organisation?

Yes

#### Please use this space to give a reason for your answer to question 11.

It has been a pleasure working with the Trial and has been informative, well run, provided scientific empirical information, pragmatic.

### SECTION 4: Lessons for the Future

#### **12.** From your experience of this Trial, what do you believe are the key lessons learned for considering potential future (re)introductions of beavers or other species?

Early and full involvement with partners and interested parties, stakeholders. listening, respecting all views and responding pragmatically and with evidence base. For beavers management options & governance are key to ensure messages and advice are consistent and appropriate

#### **13.** Following your experience of this Trial, please tell us whether or not you believe potential future (re)introductions of beavers or other species should be subject to a similar Trial process. Please give reasons for your answer.

I'd find it hard to say beaver and other species would have the same process. Beavers have much more effect on a landscape than other species and so would expect differing approaches appropriate to the species. Future re-introductions of beavers would need to be considered in the light of whether more evidence and research is required as to how much detail each trial would have in its set up. Given the level of information derived in ROBT would further trials in such detail be necessary or research needs refined to the location. The structure may not need to be the same as ROBT, for example the management group discussions would not really need to be repeated at each location for beavers.

#### **14.** Following your experience from this Trial, would you/your organisation consider participating in potential future trial (re)introductions of beavers or other species?

#### Please give reasons for your answer, including what would incentivise your participation.

Yes, refer to earlier questions regarding our reason for involvement. Redacted would likely be involved in re-introductions where the flora or fauna is dependent on an aquatic (ie riverine or wetland ) habitat.

### Section 5: Final Additional Comments

#### Please use this space to provide any additional comments you may have.

Apologies that this response has been rushed due to work pressures at the time of submission.

### Response to Findings Report

Thank for providing the opportunity to feedback on the findings report.

Looks great, Just a few notes below on the sections and some overarching thoughts here. I’m wondering if the title should be more explicit about this being recommendations/best practice/lessons learnt rather than a “review”

Across sections all I think it would be useful to recognise the need for consistency across projects, this applies in particular to management where national guidance will help in consistency and competency. Ie each project doesn’t need to re-invent the wheel. It comes over like all projects will operate in isolation when there will also be a need to have a national approach following the end of trials as animals transition into just “being there”

1 Project governance

Did the findings notes that clear timescale would be needed for reintroductions. This would seem key in order to define a start and end in order to measure if objectives are met. If an objective is to have a clear timescale then ignore this!!

Again this may be a standard “objective” but there is something about clarity in relationships of the project with other bodies working in the water environment. EG redacted had an MoU with the ROBT which was very helpful in defining roles and responsibilities.

2. Stakeholder engagement  - no additional thoughts

3. Research and monitoring programme

Not entirely clear if this is referring to ROBT only or it is referring to a national view?

4. Management Strategy - no additional thoughts other than perhaps recognising the need for national consistency and relationships between project and other regulatory bodies in the water environment

5. Public engagement - no additional thoughts

6. Broad perspectives on reintroduction trials

Not entirely sure what final sentence means (i.e. time needed for research versus a sense of urgency *for habitat recovery?* or available resource *?*).

## PARTICIPANT 9 (P9)

### SECTION 2: Your involvement in the Trial

The next three questions will ask you about which group(s) you sat on. For each one that you indicate, you will then be asked to indicate your role upon that group from a check-list.

#### **1.** Were you a member of the ROBT Steering Group (SG)?

Yes

#### Please use the following checklist to indicate your role(s) upon the Steering Group (SG). You may select as many answers as are appropriate.

Advisor (to provide advice or ideas, or to be consulted with); Informer (to inform of wider policy or developments); Informee (to be kept informed of developments)

#### **2.** Were you a member of the ROBT Beaver Management Strategy Framework Working Group (BMSF)?

Yes

#### Please use the following checklist to indicate your role(s) upon the ROBT Beaver Management Strategy Framework Working Group (BMSF).You may select as many answers as are appropriate.

Advisor (to provide advice or ideas, or to be consulted with); Informer (to inform of wider policy or developments); Informee (to be kept informed of developments)

#### **3.** Were you a member of the ROBT Science & Evidence Forum?

No

#### Please use the following checklist to indicate your role(s) upon the ROBT Science & Evidence Forum (S&E).

#### You may select as many answers as are appropriate.

*This question was not displayed to the respondent*.

#### **4.** Please state whether you/your organisation holds a particular stance on beaver reintroduction, and whether this has in any way changed over the course of the Trial?

OUr concern is with the potential impact of beaver introductions on threatened migratory salmonid populations, principally on blocking migration routed to and from spawning areas and replacement of flowing streams suited to juvenile salmonids with slow areas not suited and liable to temperature increases

#### **5.** What were the main motivations for you/your organisation to participate in the Trial?

Long-term conservation of salmonids and the socio-economically valuable anglin g they support

#### **6.** Please describe any potential risks or challenges you believe there to have been of you/your organisation's participation in the Trial.

The risk of being seen to be 'pro-beaver' rather than having objective views based on empirical evidence

### SECTION 3: Trial Review

This section will question your views on the Trial itself, and what you feel the Trial may or may not have achieved.

#### **7.** Do you consider the Trial structure to have been appropriate for conducting the Trial?

#### Please give reasons for your answer, including any changes you would suggest, if appropriate.

#### ‘Trial structure’ in this question refers to the existence of and relationships between the Licensing Group, Steering Group, Management Strategy Framework Working Group, Science & Evidence Forum, Fisheries Forum and Community Forum. It also refers to the relationship between these groups and the on-the-ground monitoring by Devon Wildlife Trust.

The structure was appropriate but the leadership was largely from groups/individuals with a pro-beaver vested interest. The various groups should have had independent chairs.

#### **8.** Do you consider the composition of organisations on the group(s) upon which you sat (SG/BMSF/S&E) to have been appropriate?

#### Please give reasons for your answer, including any changes you would suggest, if appropriate.

Yes, although there was a preponderance of vested interests (see above) and all chairs were pro-beaver

#### **9.** Please detail here if you believe there to have been any key successes of the Trial.

The main success has been to expose the issue if beaver re-introductions to a wider audience, offset the overwhelming pro-beaver position of most participants

#### **10.** Please detail here if you believe there to have been any key failures of the Trial.

Yes, a failure to focus on potential negative impacts and bias towards beaver monitoring but poor collection of evidence on impacts on fisheries

#### **11.** Overall, and on balance, have you found your experience of participating in the Trial to have been of value for you/your organisation?

Yes

#### Please use this space to give a reason for your answer to question 11.

Involvement has enabled participation in discussions and the opportunity to observe directly the lack of objectivity of the trial with its pro-beaver bias.

### SECTION 4: Lessons for the Future

#### **12.** From your experience of this Trial, what do you believe are the key lessons learned for considering potential future (re)introductions of beavers or other species?

The need to ensure that Terms of Reference produce balanced, evidence-based, objective assessments and modelling of potential impacts on existing ecosystems, with independent chairing to ensure adherence to such ToR.

#### **13.** Following your experience of this Trial, please tell us whether or not you believe potential future (re)introductions of beavers or other species should be subject to a similar Trial process. Please give reasons for your answer.

Only if the trials are truly objective and capable of being ended/reversed in the event of potential adverse impacts

#### **14.** Following your experience from this Trial, would you/your organisation consider participating in potential future trial (re)introductions of beavers or other species?

#### Please give reasons for your answer, including what would incentivise your participation.

Yes, provided the various criteria for objective trials outlined above are met

### Section 5: Final Additional Comments

#### Please use this space to provide any additional comments you may have.

## PARTICIPANT 10 (P10)

### SECTION 2: Your involvement in the Trial

The next three questions will ask you about which group(s) you sat on. For each one that you indicate, you will then be asked to indicate your role upon that group from a check-list.

#### **1.** Were you a member of the ROBT Steering Group (SG)?

No

#### Please use the following checklist to indicate your role(s) upon the Steering Group (SG). You may select as many answers as are appropriate.

*This question was not displayed to the respondent.*

#### **2.** Were you a member of the ROBT Beaver Management Strategy Framework Working Group (BMSF)?

No

#### Please use the following checklist to indicate your role(s) upon the ROBT Beaver Management Strategy Framework Working Group (BMSF).You may select as many answers as are appropriate.

*This question was not displayed to the respondent.*

#### **3.** Were you a member of the ROBT Science & Evidence Forum?

Yes

#### Please use the following checklist to indicate your role(s) upon the ROBT Science & Evidence Forum (S&E).

#### You may select as many answers as are appropriate.

Contributor - on the ground monitoring or management; Contributor - scientific research

#### **4.** Please state whether you/your organisation holds a particular stance on beaver reintroduction, and whether this has in any way changed over the course of the Trial?

My role, and that of the redacted is to undertake research to inform of the impact of beaver reintroduction and to understand the positive impacts of beaver on the landscape alongside the managment challenges that they present. This has remained consistent throughout the trial

#### **5.** What were the main motivations for you/your organisation to participate in the Trial?

Personally, to undertake research as part of my PhD research which was twinned with the trial. As an organisation, our motivation has been to gain greater understanding of beaver impacts in a landscape dominated by intensive landuse.

#### **6.** Please describe any potential risks or challenges you believe there to have been of you/your organisation's participation in the Trial.

Monitoring beavers is very challenging due to the spatially and temporally variable nature of their impacts. Designing suitable monitoring frameworks can therefore be challenging and some studies had to be altered or abandonned due to changes to beaver activity. Therefore not all of the desired investigations were completed - for example water quality/macroinvertebrate analysis has not been compled during the time frame of the trial period.

### SECTION 3: Trial Review

This section will question your views on the Trial itself, and what you feel the Trial may or may not have achieved.

#### **7.** Do you consider the Trial structure to have been appropriate for conducting the Trial?

#### Please give reasons for your answer, including any changes you would suggest, if appropriate.

#### ‘Trial structure’ in this question refers to the existence of and relationships between the Licensing Group, Steering Group, Management Strategy Framework Working Group, Science & Evidence Forum, Fisheries Forum and Community Forum. It also refers to the relationship between these groups and the on-the-ground monitoring by Devon Wildlife Trust.

Yes, I beleive the trial structure was appropriate and communication between groups was managed very well. There was enough cross over in terms of the smae people sitting on different groups to ensure that concerns/questions were raised across all relevant groups.

#### **8.** Do you consider the composition of organisations on the group(s) upon which you sat (SG/BMSF/S&E) to have been appropriate?

#### Please give reasons for your answer, including any changes you would suggest, if appropriate.

Yes I beleive that most of the relevant organisations, who represent potentially-effected stakeholders were present. It is dissapointing that some groups who have raised concerns regarding the trial were invited to sit on groups but chose not to participate.

#### **9.** Please detail here if you believe there to have been any key successes of the Trial.

The trial, for the first time in England shows that the benefits of beaver can coexist alongside appropriate managment of undesirable impacts. The structure and managment of the trial afforded generally good communication and cooperation with landowners throughout the catchment. The well organised trial also afforded us, at redacted, the opportunity to carry out research on beavers across multiple sites with good relationships with many stakeholders.

#### **10.** Please detail here if you believe there to have been any key failures of the Trial.

No I don't beleive that there were any key failures. With further funding there is ofcourse additional research that would have been extremely valuable to undertake. However, even with the massive efforts of redacted and redacted to raise money it was still necessary to prioritise certain aspects of the research namely impacts to the landscape and the subsequent impacts for landuse/landowners. If further funds had been accessed other desirable monitoring objectives including the impacts of beaver on fish could have been investigated to a greater extent. However, given the nacent state of beaver in the River Otter, monitoring these impacts may have been extremely costly whilst also being generally uninformative and therefore this was not prioritised.

#### **11.** Overall, and on balance, have you found your experience of participating in the Trial to have been of value for you/your organisation?

Yes

#### Please use this space to give a reason for your answer to question 11.

The experience has been very enjoyable and allowed me and redacted to conduct exciting, novel research into the impacts of beaver in England. We have therefore been able to make a considerabel contributon to understanding what the impact of this mammal, in an intensively managed landscape, is and could be. Much of this progress has been dependent on the affective manamgent and organisation of the trial by redacted.

### SECTION 4: Lessons for the Future

#### **12.** From your experience of this Trial, what do you believe are the key lessons learned for considering potential future (re)introductions of beavers or other species?

communication with all effected stakeholders and (as many as possible) landowners is key. If people know they can call on somebody to help if there are issues they are much more willing to take part and learn from the experience. In almost all cases, this was done extremely well during the ROBT.

#### **13.** Following your experience of this Trial, please tell us whether or not you believe potential future (re)introductions of beavers or other species should be subject to a similar Trial process. Please give reasons for your answer.

For beaver in GB it should not be necessary to go to the lengths that the ROBT went to now that the findings have been collected. The cost and time frame of similar trials would provide only limited additional information whilst slowing progress in terms of both beaver managment and introduction. That being said, I do beleive that future introductions of beaver, if they are to go ahead, should require some level of monitoring and funding to ensure that appropriate management and understanding is in place. For other native species, now extant in the UK, I beleive the ROBT provides an excellent framework on which to design reintrouction programmes.

#### **14.** Following your experience from this Trial, would you/your organisation consider participating in potential future trial (re)introductions of beavers or other species?

#### Please give reasons for your answer, including what would incentivise your participation.

Yes, the experience was wholly useful and our contributions were valued. The ability to contribute further to this type of research would be an excellent opportunity.

### Section 5: Final Additional Comments

#### Please use this space to provide any additional comments you may have.

## PARTICIPANT 11

### SECTION 2: Your involvement in the Trial

The next three questions will ask you about which group(s) you sat on. For each one that you indicate, you will then be asked to indicate your role upon that group from a check-list.

#### **1.** Were you a member of the ROBT Steering Group (SG)?

Yes

#### Please use the following checklist to indicate your role(s) upon the Steering Group (SG). You may select as many answers as are appropriate.

Advisor (to provide advice or ideas, or to be consulted with)

#### **2.** Were you a member of the ROBT Beaver Management Strategy Framework Working Group (BMSF)?

Yes

#### Please use the following checklist to indicate your role(s) upon the ROBT Beaver Management Strategy Framework Working Group (BMSF).You may select as many answers as are appropriate.

Advisor (to provide advice or ideas, or to be consulted with)

#### **3.** Were you a member of the ROBT Science & Evidence Forum?

No

#### Please use the following checklist to indicate your role(s) upon the ROBT Science & Evidence Forum (S&E).

#### You may select as many answers as are appropriate.

*This question was not displayed to the respondent*.

#### **4.** Please state whether you/your organisation holds a particular stance on beaver reintroduction, and whether this has in any way changed over the course of the Trial?

yes we do and i dont think it has. the stance is in part based on govt support and the legislative framework for which we still lack clarity on either

#### **5.** What were the main motivations for you/your organisation to participate in the Trial?

we represent the landowners and farmers on whose land beavers will provide a benefit and impact

#### **6.** Please describe any potential risks or challenges you believe there to have been of you/your organisation's participation in the Trial.

time and resources the project has had v much a conservation focus and has not been able to factor in other land management changes in the areas and what these would have meant. e.g. normalising the data this catchment is not representative and as such the results are very much related to this area

### SECTION 3: Trial Review

This section will question your views on the Trial itself, and what you feel the Trial may or may not have achieved.

#### **7.** Do you consider the Trial structure to have been appropriate for conducting the Trial?

#### Please give reasons for your answer, including any changes you would suggest, if appropriate.

#### ‘Trial structure’ in this question refers to the existence of and relationships between the Licensing Group, Steering Group, Management Strategy Framework Working Group, Science & Evidence Forum, Fisheries Forum and Community Forum. It also refers to the relationship between these groups and the on-the-ground monitoring by Devon Wildlife Trust.

yes

#### **8.** Do you consider the composition of organisations on the group(s) upon which you sat (SG/BMSF/S&E) to have been appropriate?

#### Please give reasons for your answer, including any changes you would suggest, if appropriate.

think greater involvement from local authorities especially redacted would be useful given impacts and also planning with link to net gain.

#### **9.** Please detail here if you believe there to have been any key successes of the Trial.

lots of very good science and discussion

#### **10.** Please detail here if you believe there to have been any key failures of the Trial.

this has been led and managed from an organisation with a very particular slant and as such it perhaps lack the independence that is needed. linked to this has been the funding requirement. this has meant publicity and campaigns that have been pejorative and requiring the development of "beaver connection". this makes the conversation quite led and perhaps difficult with regard to "selling" the need for parts of the management hierarchy

#### **11.** Overall, and on balance, have you found your experience of participating in the Trial to have been of value for you/your organisation?

Yes

#### Please use this space to give a reason for your answer to question 11.

it is essential farming is represented

### SECTION 4: Lessons for the Future

#### **12.** From your experience of this Trial, what do you believe are the key lessons learned for considering potential future (re)introductions of beavers or other species?

you need a truely independent body to lead it and consider all points of view. there needs to be a much more clearly articulated "out" at the start of the project. there needs to be much better understanding of the wider land management changes and the impact these would have had. i.e. what is the best tool and what provided the changes seen?

#### **13.** Following your experience of this Trial, please tell us whether or not you believe potential future (re)introductions of beavers or other species should be subject to a similar Trial process. Please give reasons for your answer.

they need to be developed but this was a very important first start

#### **14.** Following your experience from this Trial, would you/your organisation consider participating in potential future trial (re)introductions of beavers or other species?

#### Please give reasons for your answer, including what would incentivise your participation.

see answer to 11

### Section 5: Final Additional Comments

#### Please use this space to provide any additional comments you may have.

i am not sure is this is quite the space for what might be a formal response?

## PARTICIPANT 12 (P12)

### SECTION 2: Your involvement in the Trial

The next three questions will ask you about which group(s) you sat on. For each one that you indicate, you will then be asked to indicate your role upon that group from a check-list.

#### **1.** Were you a member of the ROBT Steering Group (SG)?

Yes

#### Please use the following checklist to indicate your role(s) upon the Steering Group (SG). You may select as many answers as are appropriate.

Informee (to be kept informed of developments); Other (please specify): laterly on Steering Group

#### **2.** Were you a member of the ROBT Beaver Management Strategy Framework Working Group (BMSF)?

No

#### Please use the following checklist to indicate your role(s) upon the ROBT Beaver Management Strategy Framework Working Group (BMSF).You may select as many answers as are appropriate.

*This question was not displayed to the respondent.*

#### **3.** Were you a member of the ROBT Science & Evidence Forum?

No

#### Please use the following checklist to indicate your role(s) upon the ROBT Science & Evidence Forum (S&E).

#### You may select as many answers as are appropriate.

*This question was not displayed to the respondent.*

#### **4.** Please state whether you/your organisation holds a particular stance on beaver reintroduction, and whether this has in any way changed over the course of the Trial?

interested Observer- remains unchanged until legal status is decided

#### **5.** What were the main motivations for you/your organisation to participate in the Trial?

possible value of beaver as a tool for catchment managment

#### **6.** Please describe any potential risks or challenges you believe there to have been of you/your organisation's participation in the Trial.

As a regulated business, a public service provider and a landowner there as lots of pottential risks to services and Assets from Beavers. Participation in the trail has not been challenged, but it is not widely know redacted was on the Steering group. There could be challenges from Stakeholder groups and neignbouring landowners if redacted was actively involved in particpation, rather than just observing.

### SECTION 3: Trial Review

This section will question your views on the Trial itself, and what you feel the Trial may or may not have achieved.

#### **7.** Do you consider the Trial structure to have been appropriate for conducting the Trial?

#### Please give reasons for your answer, including any changes you would suggest, if appropriate.

#### ‘Trial structure’ in this question refers to the existence of and relationships between the Licensing Group, Steering Group, Management Strategy Framework Working Group, Science & Evidence Forum, Fisheries Forum and Community Forum. It also refers to the relationship between these groups and the on-the-ground monitoring by Devon Wildlife Trust.

Structure was good for trail and sucessful outcomes resulted.

#### **8.** Do you consider the composition of organisations on the group(s) upon which you sat (SG/BMSF/S&E) to have been appropriate?

#### Please give reasons for your answer, including any changes you would suggest, if appropriate.

Yes well balanced and broad

#### **9.** Please detail here if you believe there to have been any key successes of the Trial.

-a clear plan for the future of the Otter beavers, if this is signed off by Gov. - clear steps for mitigation of beaver issues - a clear managment plan for the future - goverment awareness - increased public knowledge and particpation - better stakeholder awareness - a forum for clearing up issues as miss underatandings such as "fish eating"

#### **10.** Please detail here if you believe there to have been any key failures of the Trial.

not yet- it remains to be seen what the future of beavers in England is , so too early to say.

#### **11.** Overall, and on balance, have you found your experience of participating in the Trial to have been of value for you/your organisation?

Yes

#### Please use this space to give a reason for your answer to question 11.

informative enagaging enabled redacted to present its possetion strenghtened stakeholder relations

### SECTION 4: Lessons for the Future

#### **12.** From your experience of this Trial, what do you believe are the key lessons learned for considering potential future (re)introductions of beavers or other species?

clear planning and engagment , combined with proactive action from redacted was sucessful in this case. However- as the beavers were already there its not really a model for re-introdution as a model for how to deal with escaped and feral animals. if looking for how to do a introduction properly from the start it would not be the recomeneded approach to let the animal loose and then deal with it. Redacted have better models from thier successsfull bird reintro programmes.

#### **13.** Following your experience of this Trial, please tell us whether or not you believe potential future (re)introductions of beavers or other species should be subject to a similar Trial process. Please give reasons for your answer.

no need to repeat with Beavers once/if legal status is agreed. for other species trails coud be a good plan, dependign on species and issue.

#### **14.** Following your experience from this Trial, would you/your organisation consider participating in potential future trial (re)introductions of beavers or other species?

#### Please give reasons for your answer, including what would incentivise your participation.

yes if species was relevent to/impacted on business outcomes.

### Section 5: Final Additional Comments

#### Please use this space to provide any additional comments you may have.

## PARTICIPANT 13 (P13)

### SECTION 2: Your involvement in the Trial

The next three questions will ask you about which group(s) you sat on. For each one that you indicate, you will then be asked to indicate your role upon that group from a check-list.

#### **1.** Were you a member of the ROBT Steering Group (SG)?

Yes

#### Please use the following checklist to indicate your role(s) upon the Steering Group (SG). You may select as many answers as are appropriate.

Contributor - scientific research; Advisor (to provide advice or ideas, or to be consulted with); Reporter (lead for group output reports)

#### **2.** Were you a member of the ROBT Beaver Management Strategy Framework Working Group (BMSF)?

Yes

#### Please use the following checklist to indicate your role(s) upon the ROBT Beaver Management Strategy Framework Working Group (BMSF).You may select as many answers as are appropriate.

Contributor - scientific research; Advisor (to provide advice or ideas, or to be consulted with); Reporter (lead for group output reports)

#### **3.** Were you a member of the ROBT Science & Evidence Forum?

Yes

#### Please use the following checklist to indicate your role(s) upon the ROBT Science & Evidence Forum (S&E).

#### You may select as many answers as are appropriate.

Chair or Convenor; Contributor - scientific research; Advisor (to provide advice or ideas, or to be consulted with)

#### **4.** Please state whether you/your organisation holds a particular stance on beaver reintroduction, and whether this has in any way changed over the course of the Trial?

As a scientist I am impartial about Beaver reintroduction. As a research institute, the redacted also adopts an impartial position.

#### **5.** What were the main motivations for you/your organisation to participate in the Trial?

To undertake research, build understanding and knowledge and disseminate this knowledge to a wide range of stakeholders.

#### **6.** Please describe any potential risks or challenges you believe there to have been of you/your organisation's participation in the Trial.

It has been challenging to undertake the wide range of research that stakeholders have demanded during the trial and particularly challenging to secure enough funding to deliver all aspects of the research program that were asked for by stakeholders, including other members of the steering group. It has also been a challenge to work with certain stakeholders who either did not engage in the learning process at all or did so very late on in the trial, thus not leaving enough time to undertake research to answer their questions.

### SECTION 3: Trial Review

This section will question your views on the Trial itself, and what you feel the Trial may or may not have achieved.

#### **7.** Do you consider the Trial structure to have been appropriate for conducting the Trial?

#### Please give reasons for your answer, including any changes you would suggest, if appropriate.

#### ‘Trial structure’ in this question refers to the existence of and relationships between the Licensing Group, Steering Group, Management Strategy Framework Working Group, Science & Evidence Forum, Fisheries Forum and Community Forum. It also refers to the relationship between these groups and the on-the-ground monitoring by Devon Wildlife Trust.

Yes, I believe that the above structure was appropriate and also led to fair representation of the views of all of those groups who participated in the running of the trial.

#### **8.** Do you consider the composition of organisations on the group(s) upon which you sat (SG/BMSF/S&E) to have been appropriate?

#### Please give reasons for your answer, including any changes you would suggest, if appropriate.

Yes, I thought that the composition of the various groups was wide ranging and representative of all of the key groups that needed to be involved in the trial. It was disappointing to note that certain members of the steering group either did not participate despite being invited to every steering group meeting, or attended, but did not make contributions until is was too late for the science and evidence team to respond by undertaking research. The main recommended change here would be to compel organisations to nominate seconds to ensure attendance at meetings and further to compel each organisation to speak openly at every meeting, so as to ensure that all views were heard with enough time to then act upon them.

#### **9.** Please detail here if you believe there to have been any key successes of the Trial.

Yes, plenty. 1. Excellent partnership working across a wide range of stakeholders who did not necessarily agree about the reintroduction of beavers. 2. A great deal of research/learning that now has demonstrated a clear way forward to manage free-living beaver populations in lowland England 3. A focus on the holistic understanding of beaver reintroduction - the bigger picture than just single interests which single organisations might ordinarily just focus upon.

#### **10.** Please detail here if you believe there to have been any key failures of the Trial.

Certain groups/organisations did not take part as fully as they could have done, which was a shame, however, I would say that overall participation was very good, with just 5-10% (1 or 2 organisations) not fully participating.

#### **11.** Overall, and on balance, have you found your experience of participating in the Trial to have been of value for you/your organisation?

Yes

#### Please use this space to give a reason for your answer to question 11.

Having the opportunity to conduct research that has been co-created by a wide range of stakeholders has been a very positive experience. My feeling is that the vast majority of organisations, indeed all of those who fully participated in the trial, will also have benefit from the research that we have undertaken and indeed have indicated such in response to the science and evidence report that we have published.

### SECTION 4: Lessons for the Future

#### **12.** From your experience of this Trial, what do you believe are the key lessons learned for considering potential future (re)introductions of beavers or other species?

1. To compel, in some manner, all members of the steering group to share their ideas early and to ensure that they are bought in to the program of research that addresses the aims of the trial. Ideally this will involve all groups bringing funding and/or resources to bear, as research across 5 years on a trial of this scale is very time consuming and very costly.

#### **13.** Following your experience of this Trial, please tell us whether or not you believe potential future (re)introductions of beavers or other species should be subject to a similar Trial process. Please give reasons for your answer.

I think that the weight of science and evidence, the coherent management strategy and the conclusions of the trial suggest that running such an in-depth trial again would not be necessary. Pretty well all of the findings transfer to the vast majority of English catchments, what is needed now is a well-funded management framework for the reintroduction of beavers, based on the outcomes of the ROBT.

#### **14.** Following your experience from this Trial, would you/your organisation consider participating in potential future trial (re)introductions of beavers or other species?

#### Please give reasons for your answer, including what would incentivise your participation.

Yes, as long as it were possible to properly fund the research and evidence gathering.

### Section 5: Final Additional Comments

#### Please use this space to provide any additional comments you may have.

Overall, it has been a very positive experience working on the ROBT, probably the most enjoyable example of partnership working that I have been involved with and also the most learning that I have witnessed, at least by most organisations in a 5 year period.

## PARTICIPANT 14 (P14)

### SECTION 2: Your involvement in the Trial

The next three questions will ask you about which group(s) you sat on. For each one that you indicate, you will then be asked to indicate your role upon that group from a check-list.

#### **1.** Were you a member of the ROBT Steering Group (SG)?

Yes

#### Please use the following checklist to indicate your role(s) upon the Steering Group (SG). You may select as many answers as are appropriate.

Advisor (to provide advice or ideas, or to be consulted with)

#### **2.** Were you a member of the ROBT Beaver Management Strategy Framework Working Group (BMSF)?

No

#### Please use the following checklist to indicate your role(s) upon the ROBT Beaver Management Strategy Framework Working Group (BMSF).You may select as many answers as are appropriate.

*This question was not displayed to the respondent.*

#### **3.** Were you a member of the ROBT Science & Evidence Forum?

No

#### Please use the following checklist to indicate your role(s) upon the ROBT Science & Evidence Forum (S&E).

#### You may select as many answers as are appropriate.

*This question was not displayed to the respondent*.

#### **4.** Please state whether you/your organisation holds a particular stance on beaver reintroduction, and whether this has in any way changed over the course of the Trial?

Having been the science lead on the jointly commissioned redacted report on the feasibility of reintroducing beavers to England, I was aware that it was possible to reintroduce beavers to a great many places across England but there would be many challenges including socio-economic considerations and conflict resolution. After the publication of the feasibility report the next logical step was to carry out a detailed and carefully monitored reintroduction trial. This would provide the evidence base for whether introductions should be carried out more widely, and if so, how this could be achieved and managed. Professionally, and without affiliation to any organisation in favour or against the reintroduction of beavers, I have always taken a neutral stance and have principally been interested in the science.

#### **5.** What were the main motivations for you/your organisation to participate in the Trial?

As a scientist who has studied the behaviour, ecology and welfare of mammals, especially rodents, for many years I was attracted to the challenge of studying and learning about a potential newcomer to England, the beaver. The motivation has always been to understand the science of beaver reintroduction, the challenges of getting good data and their interpretation, i.e. evidence based conservation.

#### **6.** Please describe any potential risks or challenges you believe there to have been of you/your organisation's participation in the Trial.

None

### SECTION 3: Trial Review

This section will question your views on the Trial itself, and what you feel the Trial may or may not have achieved.

#### **7.** Do you consider the Trial structure to have been appropriate for conducting the Trial?

#### Please give reasons for your answer, including any changes you would suggest, if appropriate.

#### ‘Trial structure’ in this question refers to the existence of and relationships between the Licensing Group, Steering Group, Management Strategy Framework Working Group, Science & Evidence Forum, Fisheries Forum and Community Forum. It also refers to the relationship between these groups and the on-the-ground monitoring by Devon Wildlife Trust.

Yes, I think the trial structure has been appropriate with the various groups reporting to the main Steering group whose membership included representatives of pretty much all the main stakeholders and interested parties. Communication among the different groups was a key component and on the whole worked well.

#### **8.** Do you consider the composition of organisations on the group(s) upon which you sat (SG/BMSF/S&E) to have been appropriate?

#### Please give reasons for your answer, including any changes you would suggest, if appropriate.

Yes, I think the Steering Group meetings enabled members representing organisations with particular interests or different views on beaver reintroduction to the River Otter could voice their concerns. The paperwork for the meetings was excellent, the meetings were orderly, and well run. I also sat on the small Management group which met after the Steering Group meetings and enabled various practical actions to be considered in detail.

#### **9.** Please detail here if you believe there to have been any key successes of the Trial.

I think there are several elements that have made this trail particularly successful: 1. DWT taking the lead role and assigning staff to the trial who wereexcellent in their organisation, diligence, understanding and hard work. Equally important was that they were able to obtain the necessary funding to enable the trial to proceed - this was key. 2. The Project Delivery Plan established at the beginning was pivotal in guiding the complex set of actions that were needed, when they scheduled and whether the outputs were delivered. 3. The involvement of beaver experts (redacted) at every stage to offer critical practical guidance and advice. 4. The increasing involvement of redacted and his colleagues from Exeter University during the trial which enabled many of the scientific questions and data collection to be addressed. Others also contributed. The Science and Evidence Report is testament to how much was achieved on this front within a short period of time (in relation to beavers living in a new environment).

#### **10.** Please detail here if you believe there to have been any key failures of the Trial.

I don't think there have been any key failures. To fully understand the benefits and costs of beavers in a new environment, the trial would probably needto go on for much longer (e.g. 10-20years) and clearly this was not possible. Many of the positive and negative impacts of beavers would not be seen until population numbers reach (initially overshoot) carrying capacity. More extensive data might have been collected on some aspects of the science investigations but this is being nit-picky.

11. Overall, and on balance, have you found your experience of participating in the Trial to have been of value for you/your organisation?

Yes

#### Please use this space to give a reason for your answer to question 11.

Have found out a lot about beavers and their ecology and I have been pleased to make a contribution to this endeavour.

### SECTION 4: Lessons for the Future

#### **12.** From your experience of this Trial, what do you believe are the key lessons learned for considering potential future (re)introductions of beavers or other species?

The Beaver Management Strategy Framework is a key document that has been drawn up based on the evidence from the trial. Although directed to the future management of beavers on the River Otter it provides the framework for considering future introductions of beavers or other mammals. Although all aspects of the framework are important, I will pick out two key elements: adequate funding must be secured and detailed monitoring is an essential activity.

#### **13.** Following your experience of this Trial, please tell us whether or not you believe potential future (re)introductions of beavers or other species should be subject to a similar Trial process. Please give reasons for your answer.

Yes, trials are critically important to provide the evidence and understanding on whether reintroductions of a particular species could or should take place, and if so, how they should be carried out.

#### **14.** Following your experience from this Trial, would you/your organisation consider participating in potential future trial (re)introductions of beavers or other species?

#### Please give reasons for your answer, including what would incentivise your participation.

Yes I would - to study and learn more and offer advice if helpful.

### Section 5: Final Additional Comments

#### Please use this space to provide any additional comments you may have.

### Response to Findings Report

Obviously ROBT was a good model, though it had to think on its feet from the outset as it was reactive in nature, which would not usually be the case. I don't think many other species' introductions in the future would be so high profile (unless lynx, and maybe wildcat, come into the frame) and of course there was the rather unique backdrop of beavers in Scotland and elsewhere in the English countryside. I don't think you explicitly state anything about licensing or that a condition of the trial was that it could have been terminated under certain conditions, although this was discussed regularly and I think was a reassurance to some. I don't know whether that's important.

## PARTICIPANT 15 (P15)

### SECTION 2: Your involvement in the Trial

The next three questions will ask you about which group(s) you sat on. For each one that you indicate, you will then be asked to indicate your role upon that group from a check-list.

#### **1.** Were you a member of the ROBT Steering Group (SG)?

Yes

#### Please use the following checklist to indicate your role(s) upon the Steering Group (SG). You may select as many answers as are appropriate.

Contributor - on the ground monitoring or management

#### **2.** Were you a member of the ROBT Beaver Management Strategy Framework Working Group (BMSF)?

No

#### Please use the following checklist to indicate your role(s) upon the ROBT Beaver Management Strategy Framework Working Group (BMSF).You may select as many answers as are appropriate.

*This question was not displayed to the respondent*.

#### **3.** Were you a member of the ROBT Science & Evidence Forum?

Yes

#### Please use the following checklist to indicate your role(s) upon the ROBT Science & Evidence Forum (S&E).

#### You may select as many answers as are appropriate.

Contributor - on the ground monitoring or management

#### **4.** Please state whether you/your organisation holds a particular stance on beaver reintroduction, and whether this has in any way changed over the course of the Trial?

Pro beaver reintroduction. This has not changed over the trial.

#### **5.** What were the main motivations for you/your organisation to participate in the Trial?

Redacted one of the main partners

#### **6.** Please describe any potential risks or challenges you believe there to have been of you/your organisation's participation in the Trial.

If the trial had been stopped early DWT may have been required to trap and remove beavers from the catchment. This would have been very challenging.

### SECTION 3: Trial Review

This section will question your views on the Trial itself, and what you feel the Trial may or may not have achieved.

#### **7.** Do you consider the Trial structure to have been appropriate for conducting the Trial?

#### Please give reasons for your answer, including any changes you would suggest, if appropriate.

#### ‘Trial structure’ in this question refers to the existence of and relationships between the Licensing Group, Steering Group, Management Strategy Framework Working Group, Science & Evidence Forum, Fisheries Forum and Community Forum. It also refers to the relationship between these groups and the on-the-ground monitoring by Devon Wildlife Trust.

Yes. I think it has been appropiate for the current level of beaver activity in the River Otter catchment.

#### **8.** Do you consider the composition of organisations on the group(s) upon which you sat (SG/BMSF/S&E) to have been appropriate?

#### Please give reasons for your answer, including any changes you would suggest, if appropriate.

Yes. I think there was a broad range of organisations involved with a range of views on the reintroduction of bevers.

#### **9.** Please detail here if you believe there to have been any key successes of the Trial.

Colaboration or conservation practitioners (redacted) with academic research. Production of the BMSF, production of the Science and Evidence report, Beavers remaining in the river Otter, generating press interest and articles that show beavers can play a role in creating more flood resilient landscapes and create habitats for a wide range of biodiversity.

#### **10.** Please detail here if you believe there to have been any key failures of the Trial.

No failures but an observation. It has been a trial of the early phases of beavers recolonising a catchment with a relatively small floodplain.

#### **11.** Overall, and on balance, have you found your experience of participating in the Trial to have been of value for you/your organisation?

Yes

#### Please use this space to give a reason for your answer to question 11.

Personally I've been fortunate to have a role in the trial liaising with many different groups and working closely with the animals in the trial. I have learnt a grate deal. I think my organisation (redacted) has made a name for themselves Nationally which will have bolstered their membership (potential future financial support) and reputaion for completing high quality conservation work. I also think it's helped to further develop the connections between universites and conservation work. Getting 'proper' science done has been, and will continue to be extremely important.

### SECTION 4: Lessons for the Future

#### **12.** From your experience of this Trial, what do you believe are the key lessons learned for considering potential future (re)introductions of beavers or other species?

Many potential furture reintroductions could create measurable benefits to our environment and wider society. Exeter uni work is demonstrataing this is the case for beavers. Many landowners may be against reintroductions but attitudes can change over time if those landowners feel involved in the trial and feel that they they have support if required. Do not leave landowners to cope with reintroduced species on their own. Provide support.

#### **13.** Following your experience of this Trial, please tell us whether or not you believe potential future (re)introductions of beavers or other species should be subject to a similar Trial process. Please give reasons for your answer.

For beavers, I think that neighbouring catchments (to the River Otter) could join a management group similar to one suggested in the River Otter Beaver Management Strategy Framework. New management groups could be set up for new large catchments too. I do not think more trials are required for beavers. For other species a trial reintroduction could be useful, other species may not require such work as they may have less of an impact of surrounding landscapes.

#### **14.** Following your experience from this Trial, would you/your organisation consider participating in potential future trial (re)introductions of beavers or other species?

#### Please give reasons for your answer, including what would incentivise your participation.

Yes personally, and I'm sure redacted would particpate in future trial reintroductions. Trial reintroductions where conservation practitioners work closely with acamdemics and a broad range of interested parties is surely a good route to take.

### Section 5: Final Additional Comments

#### Please use this space to provide any additional comments you may have.

## PARTICIPANT 16 (P16)

### SECTION 2: Your involvement in the Trial

The next three questions will ask you about which group(s) you sat on. For each one that you indicate, you will then be asked to indicate your role upon that group from a check-list.

#### **1.** Were you a member of the ROBT Steering Group (SG)?

No

#### Please use the following checklist to indicate your role(s) upon the Steering Group (SG). You may select as many answers as are appropriate.

*This question was not displayed to the respondent*.

#### **2.** Were you a member of the ROBT Beaver Management Strategy Framework Working Group (BMSF)?

No

#### Please use the following checklist to indicate your role(s) upon the ROBT Beaver Management Strategy Framework Working Group (BMSF).You may select as many answers as are appropriate.

*This question was not displayed to the respondent*.

#### **3.** Were you a member of the ROBT Science & Evidence Forum?

Yes

#### Please use the following checklist to indicate your role(s) upon the ROBT Science & Evidence Forum (S&E).

#### You may select as many answers as are appropriate.

Contributor - scientific research

#### **4.** Please state whether you/your organisation holds a particular stance on beaver reintroduction, and whether this has in any way changed over the course of the Trial?

I can't speak for others in the organisation, but personally, I was very pro-beaver reintroduction to England prior to involvement in the ROBT. I would say Istill am, although feel I have a greater appreciation of the challenges and conflicts that beaver reintroduction can have. While I believe that, on balance, the overall benefits associated with reintroducing beavers out weight the costs, I am more understanding to the fact that in some locations / scenarios (i.e. at smaller more site specific scales) this may not always be the case.

#### **5.** What were the main motivations for you/your organisation to participate in the Trial?

I was personally motivated because it represented an opportunity to conduct field work on a really exciting ecological / conservation topic and one thatwas of genuine interest to me. It also provided an opportunity to work alongside other research groups (redacted) and organisations (redacted), which I enjoy. From an organisation perspective, I suspect the reasons are similar, but also include the fact it related to existing research that was being conducted within the group, aligned with our areas of expertise and was an opportunity to continue research on the topic.

#### **6.** Please describe any potential risks or challenges you believe there to have been of you/your organisation's participation in the Trial.

None that relate to the fact that beaver reintroduction to England is quite a politically charged topic.

### SECTION 3: Trial Review

This section will question your views on the Trial itself, and what you feel the Trial may or may not have achieved.

#### **7.** Do you consider the Trial structure to have been appropriate for conducting the Trial?

#### Please give reasons for your answer, including any changes you would suggest, if appropriate.

#### ‘Trial structure’ in this question refers to the existence of and relationships between the Licensing Group, Steering Group, Management Strategy Framework Working Group, Science & Evidence Forum, Fisheries Forum and Community Forum. It also refers to the relationship between these groups and the on-the-ground monitoring by Devon Wildlife Trust.

The various Groups / Forums seemed well structured in terms of representing a variety of stakeholders or being focused on specific aspects (science vs. management for example). I also get the impression that DWT put in a lot of work to liaise with a wide variety of audiences on a more ad-hoc basis. Whether the latter is something that can be sustained indefinitely, or in other regions of the country as more beaver populations are reintroduced in the future will be interesting to see. I was also a little unclear whether there was any hierarchical structure the the groups or what the lines of feedback were. For example, could the Management working group identify questions that could be fed back to the Science and Evidence Forum and influence the direction of research? The Community and Fisheries Forums seemed useful for providing updates to stakeholders on current status / research, but could (or did) feedback from these groups influence the programme of research (I imagine it would be difficult to be too reactive with an already outlined programme of research, with resources allocated). The above comment likely related to my relatively limited involvement and as numerous people were involved in multiple working groups, I assume there were clear lines of communication between them.

#### **8.** Do you consider the composition of organisations on the group(s) upon which you sat (SG/BMSF/S&E) to have been appropriate?

#### Please give reasons for your answer, including any changes you would suggest, if appropriate.

Yes. Representation struck a good balance between those with an interest in the topic (Community / Fisheries Forums) and those that were better placed to guide, advise or conduct management / research activities. I'm sure keeping these groups separate and limited greatly aided the overall coordination and management of the trial.

#### **9.** Please detail here if you believe there to have been any key successes of the Trial.

I think the ultimate success of the trial has been the high quality research that has been conducted. It has provided an evidence base for decision makingand highlighted areas where more work is needed. At the same time DWT have done a great job with conflict management and I'm sure many lessons have been learnt for the future.

#### **10.** Please detail here if you believe there to have been any key failures of the Trial.

As my levels of involvement in the trial were relatively low, I'm not really aware of (or feel there are) any key failures. I think it is impossible to research every aspect relating to beaver reintroductions in a 5 year trial period, and what has been done is pretty substantial. Time and resources are limited, and so there will always remain question marks over numerous topics. Those with particular interests in these topics may well feel that a lack of information = failure. What is important is that research continues where it is needed (and that this is well resourced), and that management of beavers is adaptive, and driven by current evidence as well as that which emerges in the future.

#### **11.** Overall, and on balance, have you found your experience of participating in the Trial to have been of value for you/your organisation?

Yes

#### Please use this space to give a reason for your answer to question 11.

Very much enjoyed the research elements I was involved in and the topic is fascinating to me. It was also very interesting to be part of Forums where you hear a range of perspectives / opinions. It helped reaffirm the need for evidence based opinions (on all sides of the debate).

### SECTION 4: Lessons for the Future

#### **12.** From your experience of this Trial, what do you believe are the key lessons learned for considering potential future (re)introductions of beavers or other species?

Proactively engage with a wide variety of stakeholders, being open, honest and taking opinions / concerns into account. Resources (personnel, time, money) need to be allocated for managing potential conflicts, and manage needs to be reactive as many of the issue are likely unforeseen. Balancing the need for a clear programme of research with the value of being able to be reactive so research can focus on areas that emerge as being of key importance or lacking in existing evidence. The need for realistic expectations of what can be achieved during a relatively short trial reintroduction (perhaps particularly the expectations of various stakeholders). I think the research that has been done is great, but really there is scope for it to continue for decades. What is important is that it is well resourced and that the management of beavers is adaptive to the new evidence that emerges.

#### **13.** Following your experience of this Trial, please tell us whether or not you believe potential future (re)introductions of beavers or other species should be subject to a similar Trial process. Please give reasons for your answer.

I believe they should. Trials such as this are invaluable for providing context specific evidence. The caveat is that it will not be possible to answer all thequestions within the scope of such trials. The end of these kinds of trials does not indicate that no more research is required. They provide a starting point for decision making and management and this should be made clear to all stakeholders involved.

#### **14.** Following your experience from this Trial, would you/your organisation consider participating in potential future trial (re)introductions of beavers or other species?

Yes

#### Please give reasons for your answer, including what would incentivise your participation.

Yes. Involvement needs to be adequately resourced.

### Section 5: Final Additional Comments

#### Please use this space to provide any additional comments you may have.

## PARTICIPANT 17 (P17)

### SECTION 2: Your involvement in the Trial

The next three questions will ask you about which group(s) you sat on. For each one that you indicate, you will then be asked to indicate your role upon that group from a check-list.

#### **1.** Were you a member of the ROBT Steering Group (SG)?

Yes

#### Please use the following checklist to indicate your role(s) upon the Steering Group (SG). You may select as many answers as are appropriate.

Contributor - on the ground monitoring or management; Advisor (to provide advice or ideas, or to be consulted with); Informee (to be kept informed of developments)

#### **2.** Were you a member of the ROBT Beaver Management Strategy Framework Working Group (BMSF)?

Yes

#### Please use the following checklist to indicate your role(s) upon the ROBT Beaver Management Strategy Framework Working Group (BMSF).You may select as many answers as are appropriate.

Contributor - on the ground monitoring or management; Advisor (to provide advice or ideas, or to be consulted with); Informer (to inform of wider policy or developments); Informee (to be kept informed of developments)

#### **3.** Were you a member of the ROBT Science & Evidence Forum?

No

#### Please use the following checklist to indicate your role(s) upon the ROBT Science & Evidence Forum (S&E).

#### You may select as many answers as are appropriate.

*This question was not displayed to the respondent*.

#### **4.** Please state whether you/your organisation holds a particular stance on beaver reintroduction, and whether this has in any way changed over the course of the Trial?

Redacted welcomes reintroductions of native species provided they follow IUCN guidelines. We do, however, take a slightly cautious approach because we believe that reintroductions often make light of the mitigation and control issues that are likely to arise if they are successful. None of this has changed as a result of ROBT

#### **5.** What were the main motivations for you/your organisation to participate in the Trial?

Believeing that beaver reintroducion would be successful, we were keen to try to ensure that future management and protection status would be as simple as possible. We were also keen to ensure that proper account was taken if likely negative impacts, especially on migratory fish.

#### **6.** Please describe any potential risks or challenges you believe there to have been of you/your organisation's participation in the Trial.

many redacted members would not welcome the return of beavers. We needed to explain why we were involved to member groups, and to reassure them that we would take account of their views in making our contribution to the trial.

### SECTION 3: Trial Review

This section will question your views on the Trial itself, and what you feel the Trial may or may not have achieved.

#### **7.** Do you consider the Trial structure to have been appropriate for conducting the Trial?

#### Please give reasons for your answer, including any changes you would suggest, if appropriate.

#### ‘Trial structure’ in this question refers to the existence of and relationships between the Licensing Group, Steering Group, Management Strategy Framework Working Group, Science & Evidence Forum, Fisheries Forum and Community Forum. It also refers to the relationship between these groups and the on-the-ground monitoring by Devon Wildlife Trust.

Overall I feel that this structure worked well, although I think that the possible negative impacts on fish were somewhat glossed over.

#### **8.** Do you consider the composition of organisations on the group(s) upon which you sat (SG/BMSF/S&E) to have been appropriate?

#### Please give reasons for your answer, including any changes you would suggest, if appropriate.

The composition of the two groups that I was involved with seemed fine to me.

#### **9.** Please detail here if you believe there to have been any key successes of the Trial.

People and organisations with a wide and differing range of viewpoints worked constructively together.

#### **10.** Please detail here if you believe there to have been any key failures of the Trial.

I believe that the evidence of likely negative impacts on migratory fish was made light of, both in meetings and reports.

#### **11.** Overall, and on balance, have you found your experience of participating in the Trial to have been of value for you/your organisation?

Yes

#### Please use this space to give a reason for your answer to question 11.

While redacted may not have agreed with all of the final outcomes, there were no major surprises. We were also able to report back to colleagues and members within perfectly acceptable confidentiality constraints.

### SECTION 4: Lessons for the Future

#### **12.** From your experience of this Trial, what do you believe are the key lessons learned for considering potential future (re)introductions of beavers or other species?

Beavers are great colonists, and will successfully recolonise much of the UK. But, they have the potential to cause very serious and expensive damage in many areas, so control must be simple and not heavily bound up in "red tape". Many people who are enthusiastic about beavers fail to properly recognise that. Similar issues apply with the likes of wild boar and pine martens.

#### **13.** Following your experience of this Trial, please tell us whether or not you believe potential future (re)introductions of beavers or other species should be subject to a similar Trial process. Please give reasons for your answer.

Trials of this kind are expensive, both for the organisation in charge, and participating organisations. So, a detailed trial like this for each beaver reintroduction would be more than is needed, but for other species there could be value in a similar approach. I fear, for example, that beyond calling the odd meeting, those who will have to carry the damage and cost caused by pine martens have had little say so far.

#### **14.** Following your experience from this Trial, would you/your organisation consider participating in potential future trial (re)introductions of beavers or other species?

#### Please give reasons for your answer, including what would incentivise your participation.

Yes. In general our members would expect us to do so.

### Section 5: Final Additional Comments

#### Please use this space to provide any additional comments you may have.

This survey is a good idea.

### Response to Findings Report

Thanks for this useful summary. I think you have captured the issues well. As you know, I have been concerned all along that we have glossed over the potential damaging impacts on fish migration. I think that a key point in regard to that is that all fish are not equal. There has been a tendency to lump them together in a slightly naive way, rather than consider impacts on individual species and even subspecies. For example, impeding the migration of sea trout while improving insect availability for brown trout does not necessarily balance out.

I’m glad that you mention fish as a specific example in your first bullet under section 6, and I hope you will be able to flesh this out in greater detail in the peer reviewed report. I hope also that you will be able to use this as an example to warn against brushing issues like this aside, in terms of maintaining stakeholder engagement through the trial process.

## PARTICIPANT 18 (P18)

### SECTION 2: Your involvement in the Trial

The next three questions will ask you about which group(s) you sat on. For each one that you indicate, you will then be asked to indicate your role upon that group from a check-list.

#### 1. Were you a member of the ROBT Steering Group (SG)?

Yes

#### Please use the following checklist to indicate your role(s) upon the Steering Group (SG). You may select as many answers as are appropriate.

Contributor – on the ground monitoring or management; Contributor – scientific research; Advisor (to provide advice or ideas, or to be consulted with)

#### 2. Were you a member of the ROBT Beaver Management Strategy Framework Working Group (BMSF)?

Yes

#### Please use the following checklist to indicate your role(s) upon the ROBT Beaver Management Strategy Framework Working Group (BMSF).You may select as many answers as are appropriate.

Contributor – on the ground monitoring or management; Contributor – scientific research; Advisor (to provide advice or ideas, or to be consulted with)

#### 3. Were you a member of the ROBT Science & Evidence Forum?

No

#### Please use the following checklist to indicate your role(s) upon the ROBT Science & Evidence Forum (S&E).

#### You may select as many answers as are appropriate.

*This question was not displayed to the respondent*.

#### 4. Please state whether you/your organisation holds a particular stance on beaver reintroduction, and whether this has in any way changed over the course of the Trial?

Pro-beaver reintroduction, hasnt changed

#### 5. What were the main motivations for you/your organisation to participate in the Trial?

Working directly with beaver reintroduction and research for last 12 years in Scotland and wish to see the species officially and responsibly restored across Britain.

#### 6. Please describe any potential risks or challenges you believe there to have been of you/your organisation's participation in the Trial.

No risks. Similar animal and people management / engagement challenges to beaver restoration, though sometimes articulating that these would be highly similar in the south, especially over time, was sometimes difficult

### SECTION 3: Trial Review

This section will question your views on the Trial itself, and what you feel the Trial may or may not have achieved.

#### **7.** Do you consider the Trial structure to have been appropriate for conducting the Trial?

#### Please give reasons for your answer, including any changes you would suggest, if appropriate.

#### ‘Trial structure’ in this question refers to the existence of and relationships between the Licensing Group, Steering Group, Management Strategy Framework Working Group, Science & Evidence Forum, Fisheries Forum and Community Forum. It also refers to the relationship between these groups and the on-the-ground monitoring by Devon Wildlife Trust.

#### **8.** Do you consider the composition of organisations on the group(s) upon which you sat (SG/BMSF/S&E) to have been appropriate?

#### Please give reasons for your answer, including any changes you would suggest, if appropriate.

#### **9.** Please detail here if you believe there to have been any key successes of the Trial.

Public engagement and support, got a licence and beavers allowed to stay! Also generally good communications, wide stakeholder engagement, high quality science, well organised campaign – good social media

#### **10.** Please detail here if you believe there to have been any key failures of the Trial.

Not establishing a genetically diverse population – more opportunities for actual monitoring of animals themselves – science very impact focused, potentially promoting a more expensive future management strategy – should be more cost effective and efficient, sometimes feel holding up beaver as a special species requiring special/expansive measure from now on – again normalise the species

#### **11.** Overall, and on balance, have you found your experience of participating in the Trial to have been of value for you/your organisation?

#### Please use this space to give a reason for your answer to question 11.

Enabled us to undertake a suite of research into a wild living beaver population in a modern intensively managed British landscape.

### SECTION 4: Lessons for the Future

#### **12.** From your experience of this Trial, what do you believe are the key lessons learned for considering potential future (re)introductions of beavers or other species?

Stremline, not be overly cautious, public engagement is key, greater aspirations on numbers and scale, learn from other projects/experiences to build on knowledge, don’t reinvent the wheel, take more things as red with confidence

#### **13.** Following your experience of this Trial, please tell us whether or not you believe potential future (re)introductions of beavers or other species should be subject to a similar Trial process. Please give reasons for your answer.

This type of trial set-up has a time and place but is not required going forward for every beaver project/catchment given resource investment - time and costs. Stakeholder engagement and facilitating communications and information dissemination is vital for reintroduction projects but I think we are in danger this sets a precedence that conservation translocations require this level of investment every time, when other similar sectors have no such requirement or expectations there off e.g. animal release for shooting etc. The whole process needs to become normalised and streamlined, especially given current expected recession concerns, we need to be careful we aren’t holding up reintroductions as overly complicated and expensive and therefore subject to continua scrutiny

#### **14.** Following your experience from this Trial, would you/your organisation consider participating in potential future trial (re)introductions of beavers or other species?

#### Please give reasons for your answer, including what would incentivise your participation.

Yes – would be keen to have scientific and protocol design input, along with practical animal monitoring/husbandry/ management aspects

### Section 5: Final Additional Comments

#### Please use this space to provide any additional comments you may have.

## PARTICIPANT 19 (P19)

### SECTION 2: Your involvement in the Trial

The next three questions will ask you about which group(s) you sat on. For each one that you indicate, you will then be asked to indicate your role upon that group from a check-list.

#### **1.** Were you a member of the ROBT Steering Group (SG)?

No

#### Please use the following checklist to indicate your role(s) upon the Steering Group (SG). You may select as many answers as are appropriate.

*This question was not displayed to the respondent*.

#### **2.** Were you a member of the ROBT Beaver Management Strategy Framework Working Group (BMSF)?

Yes

#### Please use the following checklist to indicate your role(s) upon the ROBT Beaver Management Strategy Framework Working Group (BMSF).You may select as many answers as are appropriate.

Contributor - on the ground monitoring or management; Advisor (to provide advice or ideas, or to be consulted with); Informer (to inform of wider policy or developments); Informee (to be kept informed of developments); Other (please specify): Landowner with management interests

#### **3.** Were you a member of the ROBT Science & Evidence Forum?

Yes

#### Please use the following checklist to indicate your role(s) upon the ROBT Science & Evidence Forum (S&E).

#### You may select as many answers as are appropriate.

Contributor - on the ground monitoring or management; Advisor (to provide advice or ideas, or to be consulted with); Informee (to be kept informed of developments)

#### **4.** Please state whether you/your organisation holds a particular stance on beaver reintroduction, and whether this has in any way changed over the course of the Trial?

Our stance has remained unchanged. Cautiously supportive as long as management considerations are addressed. We feel they have been as much as the Trial will allow. We are obviously better informed than at the start, but to be honest weren't poorly informed to begin with. It has largely played out as we expected.

#### **5.** What were the main motivations for you/your organisation to participate in the Trial?

Influence the outcome - particularly related to management approach. We saw early on that beavers were in England and expanding and that there was no political will to ever remove them. Thus, in our view better to accept this and be on the inside influencfing the process to get the best outcomes for beavers and land managers than grumbling from the sidelines.

#### **6.** Please describe any potential risks or challenges you believe there to have been of you/your organisation's participation in the Trial.

Risks - we will have long-term management costs and liabilities related to beaver management when the Trial is completed Challenges- making sure that mangement considerayons were taken seriously, but to be fair, there was broad acceptance of this point early on from all parties so it wasn't really a challenge Challenges - the public automatically thinking we are anti-nbeaver because we are a landowner. There remains a degree of polarisation in public perceoption on thsi point which is not helpful We remain nervous as to teh degree to which there will be support for beaver management post Trial. However, we accept that the Trial hs done all it reasonaby can so are happy with that.

### SECTION 3: Trial Review

This section will question your views on the Trial itself, and what you feel the Trial may or may not have achieved.

#### **7.** Do you consider the Trial structure to have been appropriate for conducting the Trial?

#### Please give reasons for your answer, including any changes you would suggest, if appropriate.

#### ‘Trial structure’ in this question refers to the existence of and relationships between the Licensing Group, Steering Group, Management Strategy Framework Working Group, Science & Evidence Forum, Fisheries Forum and Community Forum. It also refers to the relationship between these groups and the on-the-ground monitoring by Devon Wildlife Trust.

Yes very happy. It worked well in our view and covered all bases and interests. Relationshsips were good and our relationship with redacted and others parties is stronger than at teh beginning.

#### **8.** Do you consider the composition of organisations on the group(s) upon which you sat (SG/BMSF/S&E) to have been appropriate?

#### Please give reasons for your answer, including any changes you would suggest, if appropriate.

Yes.

#### **9.** Please detail here if you believe there to have been any key successes of the Trial.

Listening, treating all concerns seriously, trust and good communication

#### **10.** Please detail here if you believe there to have been any key failures of the Trial.

No

#### **11.** Overall, and on balance, have you found your experience of participating in the Trial to have been of value for you/your organisation?

Yes

#### Please use this space to give a reason for your answer to question 11.

### SECTION 4: Lessons for the Future

#### **12.** From your experience of this Trial, what do you believe are the key lessons learned for considering potential future (re)introductions of beavers or other species?

Clear purpose and objectives; good information based on science; clear governance structure; broad consultation; taking the time to communicate; delivering on promises; listening to concerns; building good relationships

#### **13.** Following your experience of this Trial, please tell us whether or not you believe potential future (re)introductions of beavers or other species should be subject to a similar Trial process. Please give reasons for your answer.

Depends on the species. Certainly mangement groups and risk assessment/feasibility study are required for other catchments as they expand. No need for a Trial as I believe we have moved on from this. For other species it depends on the potential ecological/economic impact. Probably worth doing something similar for pine martins (for example) but less complicated as already a native species. Probably not necesary for species that are 'insignificant' in terms of impact (such as water voles) BUT, you woudl still need good science behind it otherwise reintroduction unlikley to b successful,. Follow IUCN guidelines.

#### **14.** Following your experience from this Trial, would you/your organisation consider participating in potential future trial (re)introductions of beavers or other species?

#### Please give reasons for your answer, including what would incentivise your participation.

Yes. Beavers now considered business as usual. Incentives to participate in fuiture reintroductions? Any species where a compelling argument for the benefits of their (re)introduction can be made. We are not against anythig in principle.

### Section 5: Final Additional Comments

#### Please use this space to provide any additional comments you may have.

## PARTICIPANT 20 (P20)

***NB.*** *Please note details on P20 under ‘Participants and Survey Distribution’ (under ’Methods’) in the main text for additional notes on P20.*

### SECTION 2: Your involvement in the Trial

The next three questions will ask you about which group(s) you sat on. For each one that you indicate, you will then be asked to indicate your role upon that group from a check-list.

#### **1.** Were you a member of the ROBT Steering Group (SG)?

No

#### Please use the following checklist to indicate your role(s) upon the Steering Group (SG). You may select as many answers as are appropriate.

*This question was not displayed to the respondent.*

#### **2.** Were you a member of the ROBT Beaver Management Strategy Framework Working Group (BMSF)?

No

#### Please use the following checklist to indicate your role(s) upon the ROBT Beaver Management Strategy Framework Working Group (BMSF).You may select as many answers as are appropriate.

*This question was not displayed to the respondent.*

#### **3.** Were you a member of the ROBT Science & Evidence Forum?

No

#### Please use the following checklist to indicate your role(s) upon the ROBT Science & Evidence Forum (S&E).

#### You may select as many answers as are appropriate.

*This question was not displayed to the respondent.*

#### **4.** Please state whether you/your organisation holds a particular stance on beaver reintroduction, and whether this has in any way changed over the course of the Trial?

Neutral at start; against unrestricted introductions at the end

#### **5.** What were the main motivations for you/your organisation to participate in the Trial?

To represent the interests of the fish and river's ecology given the introduction of a new species.

#### **6.** Please describe any potential risks or challenges you believe there to have been of you/your organisation's participation in the Trial.

Our participation was in part unsatisfactory: promises about our ability to comment on trial outputs set out in the TOR of the Fishery Forum were never met. The trial was not always open to challenge/debate. Meetings were not productive (they tended to be filled with presentations about the work of the trial and how good beavers were, with little or no time for debate)

### SECTION 3: Trial Review

This section will question your views on the Trial itself, and what you feel the Trial may or may not have achieved.

#### **7.** Do you consider the Trial structure to have been appropriate for conducting the Trial?

#### Please give reasons for your answer, including any changes you would suggest, if appropriate.

#### ‘Trial structure’ in this question refers to the existence of and relationships between the Licensing Group, Steering Group, Management Strategy Framework Working Group, Science & Evidence Forum, Fisheries Forum and Community Forum. It also refers to the relationship between these groups and the on-the-ground monitoring by Devon Wildlife Trust.

See comments at Para 6 on how the work of the Fisheries Forum was of limited value and that the Forum was not given the promised opportunity to comment on other parts of the trial's outputs pre-publication.

#### **8.** Do you consider the composition of organisations on the group(s) upon which you sat (SG/BMSF/S&E) to have been appropriate?

#### Please give reasons for your answer, including any changes you would suggest, if appropriate.

The Fisheries Forum was unwieldy due to an attempt to involve everyone. Various important national bodies were invited to attend but failed to do so and so the wider perspective was missing. A more selective choice and proper attendance would have helped.

#### **9.** Please detail here if you believe there to have been any key successes of the Trial.

The fact that it got done and did at least try to quantify some of the issues (but see next question too)

#### **10.** Please detail here if you believe there to have been any key failures of the Trial.

The trial was marred by a number of issues: pre-existing pro/anti-beaver agendas, most notably the enthusiasm of many 'researchers' for beavers; the financial issues e.g. generating public support and fund raising by emphasising pro-beaver stories rather than balance of argument; involvement of 'researchers' and parent organisations in pro-beaver PR campaigns and financial gain from them; the relatively short time scale and superficiality of the research; the lack of baselines and control studies; the interference of external bodies with pro-beaver agendas. The trial has failed to answer many questions, in particular what effect larger free-ranging populations of beavers will have in the longer term on tree loss and on migratory fish.

#### **11.** Overall, and on balance, have you found your experience of participating in the Trial to have been of value for you/your organisation?

No

#### Please use this space to give a reason for your answer to question 11.

It has been good and bad. The trial personalities have in large part tried to please everyone and of course that has been difficult where opinions differ. They were under great pressure as well of course, given the number of competing agendas and limited resources. That said our involvement was unsatisfactory as in the end the Fisheries Forum TOR which we all signed off were never fulfilled. Plus we do not feel the trial reached fully justified conclusions.

### SECTION 4: Lessons for the Future

#### **12.** From your experience of this Trial, what do you believe are the key lessons learned for considering potential future (re)introductions of beavers or other species?

1. There should be properly funded (meaning not funded by the organisations involved generating PR for one outcome). 2. They need to be externally monitored and rigorously scientific (proper controls and baselines in place before you start for example) 3. A very careful and pre-cautionary principle should always be applied.

#### **13.** Following your experience of this Trial, please tell us whether or not you believe potential future (re)introductions of beavers or other species should be subject to a similar Trial process. Please give reasons for your answer.

As above this trial was an inadequate basis upon which to allow a free-ranging uncontrolled population of beavers in the UK. Trials like this require a more rigorous and impartial approach and should only be allowed to conclude what is fully justified, not what their controllers wish to see.

#### **14.** Following your experience from this Trial, would you/your organisation consider participating in potential future trial (re)introductions of beavers or other species?

#### Please give reasons for your answer, including what would incentivise your participation.

Yes in the hope that more rigorous trials can take place.

### Section 5: Final Additional Comments

#### Please use this space to provide any additional comments you may have.

The trial was in part successful and well-run, but it should be aware of its errors and omissions and not exaggerate what it achieved in order to push forward a pro-beaver agenda. The conflicts of interest are clear and our worry is that in the longer term we may well come to regret decisions based on this trial.
