# Supplementary material for: Renewed coexistence: learning from steering group stakeholders on a beaver reintroduction project in England
Source: Eur J Wildl Res. 2021 Dec 3;68(1):1. doi: 10.1007/s10344-021-01555-6 (PMC8640482; doi:10.1007/s10344-021-01555-6)
Supplement: Supplementary file 2 — Supplementary file2 (DOCX 26 KB) [file 10344_2021_1555_MOESM2_ESM.docx]

# SUPPORTING INFORMATION: Findings Report as was Shared with Participants

**Article**: Renewed Coexistence: Learning from Steering Group Stakeholders on a Beaver Reintroduction Project in England

**Journal**: European Journal of Wildlife Research

**Authors**: Roger E Auster (University of Exeter; [r.e.auster@exeter.ac.uk](mailto:r.e.auster@exeter.ac.uk)), Prof. Stewart Barr (University of Exeter), Prof. Richard Brazier (University of Exeter)

# research Findings Report: ‘Review’ of the River Otter Beaver Trial

## research reminder

Following a proposal to the ROBT Steering Group meeting on 13th February 2020, the University of Exeter has conducted research into the experiences of Steering Group members in the River Otter Beaver Trial processes. The research aim was to explore experiences and views of the ROBT process, and to identify key factors that may help to inform future processes or decisions in other potential (re)introduction projects.

The invitation to participate was shared with all members of the Steering Group and, at the request of the Steering Group at the February meeting, also with the members of both the Beaver Management Strategy Framework Working Group and Science & Evidence Forum as the leads for key ROBT document outputs.

Participation involved completing an online questionnaire between 30^th^ April and the extended deadline of 10^th^ August 2020.

## about this report

At the time of the questionnaire, we promised participants the opportunity to view a findings report prior to publication of results. As such, this findings report provides an overview summary of key points from participant responses, identified through a traditional qualitative thematic analysis.

The aim of this report is to provide you, as a participant, with an opportunity for any further comment prior to publication of the findings.

## next steps

Please return any comments by email to [rea213@exeter.ac.uk](mailto:rea213@exeter.ac.uk) by **Monday 21^st^ December 2020**.

In accordance with the research information provided prior to your participation, this research is currently being written up as a full manuscript to be submitted to the scientific review process. This is to ensure appropriate method and objectivity prior to results publication. This will also be included in a PhD thesis.

The manuscript will incorporate the key points outlined below. This findings report provides you with the opportunity to comment prior to the submission for peer review. In order to meet the submission deadline, it will not be possible to address any comments received after the 21^st^ December.

As was promised at the time of participation, all participant comments will remain anonymised in any research output. Any comments provided in response to this report will continue to be treated as anonymous.

Following scientific peer review, published work will be shared back with you.

As these findings have not yet been peer reviewed, please treat this report as **confidential**. [**NB.** This was the case at the time of sharing the Findings Report]

## a thank you

I would like to express a personal thank you for your contribution towards this work. I hope you will find it both interesting and of value. I would also like to take this opportunity to send you my best wishes for the upcoming holiday period.

## KEY points

The following is a summary of key points that have been identified. These will be discussed more fully in the final manuscript, with anonymised evidential quotations from research participants.

In this report, the key points are outlined under six key headings:

[1) Project Governance](#_1._Project_governance)

[2) Stakeholder Engagement](#_2._stakeholder_engagement)

[3) Research and Monitoring Programme](#_3._research_and)

[4) Management Strategy](#_4._management_strategy)

[5) Public Engagement](#_5._Public_engagement)

[6) Broad Perspectives on Reintroduction Trials](#_6._broad_perspectives)

#### 1. Project governance

- *Project Aims -* Reintroduction projects should have clearly defined objectives and governance. Objectives need to be feasible within the Trial scope, and expectations will need to be managed accordingly.
- *Leadership -* Reintroductions require committed leadership. There will need to be an honest and transparent approach, recognising both benefits and conflicts. One consideration may be for an independent and impartial chair for stakeholder groups. If this is not possible, transparency will increase in importance to maintain trust between parties.
- *Structure -* Trial frameworks (or group remits) will need to be clearly defined and articulated, enabling the project aims to be met whilst avoiding duplication of effort.
- *Resourcing -* Trials of the scale of the ROBT require financial, time and personnel resource. This can include financial risk for the project leads (eg. in the event negative beaver impacts incurred costs).

#### 2. stakeholder engagement

- *Outreach -* Stakeholder engagement was significant in the ROBT with an active effort to ensure the range of key interests were represented. Future projects should also seek to identify and engage with a full range of stakeholders.
- *Respectful, Constructive Discussion -* Respectful, partnership working was key to learning from one another and resolving areas of disagreement.
- *Challenges* - There can be challenges associated with stakeholder engagement including: stakeholder willingness to participate; potential risk of partnership breakdown; reputational risk for participating stakeholders (eg. risk of being perceived to hold a stance); resources may be required for participation (eg. staff time).
- *Value of Participation for Stakeholders -* Trial participation was mostly reported to be of value for the individuals/organisations involved, and there was willingness amongst participants from the three groups to participate in potential future reintroduction trials, though for some this was conditional on certain factors (eg. if stakeholder interests were affected, or objectivity in the approach).

#### 3. research and monitoring programme

- *Broad Reception -* Reintroduction trials will require a well-planned scientific research and monitoring programme, ideally co-created with stakeholders and reactive to emerging issues.
- *Programme Focus -* The programme should consider a holistic understanding of reintroduction-related issues, whilst building on learning from previous experience or research elsewhere.
- *Feasibility Limitations -* There may be challenges which limit the feasibility of what research can be achieved, including financial, physical, or temporal limitations. These will need to be accounted for in strategic decision-making, the management of stakeholder expectations, and amongst the expectations stakeholders hold of a project’s outcomes.
- *Objectivity -* Objectivity in the research programme will help to maintain trust with stakeholders.

#### 4. management strategy

- *Strategy Required –* Reintroduction projects will require a management plan for potential conflict scenarios, with resources allocated for conflict management.
- *When to Intervene –* Proactive intervention is beneficial where possible, but management may also need to be quick and reactive to emerging issues that cannot be addressed in advance.
- *Clearly Communicated* – The management plan should be clear and accessible, with those affected knowing that support is available.
- *Long Term Thinking* – As well as an in-trial management strategy, consideration should be given to long-term management in case the species permanently remains. Some decisions in the long term may not be in the hands of project leads or stakeholders (eg government legislation) but learning can be provided or cross-partnership recommendations made (eg BMSF document).

#### 5. Public engagement

- *Importance of* *Public Engagement -* Public Engagement provides opportunities: to educate the public; provide knowledge of available management support; foster coexistence with the reintroduced species; provide knowledge of available management support; garner public support or to express contrasting viewpoints.
- *Reach and Tone –* Public Engagement will require a broad reach. A balanced presentation of evidence can facilitate better engagement with groups of differing perspectives.

#### 6. broad perspectives on reintroduction trials

- *ROBT as a Model* – Future reintroduction projects could learn from and build upon the ROBT framework. Reintroduction trials provide opportunity to learn and build evidence for decision-making, although learning is likely to continue beyond a trial’s conclusion, particularly on outstanding research questions (eg. the relationship between beavers and fish). Some respondents felt that future trials should have plans in place prior to any species release, rather than being set up reactively to unlicensed releases.
- *Species Variance* – Although reintroduction trials were broadly a supported process here, it is suggested that future trials may not need to be of the same scale for species other than beavers, particularly species that may not have as much impact on the wider landscape.
- *Scale, Duration and Species Population Decisions* – Reintroduction trials will require decisions with some likely trade-offs. These include on: the level of cautiousness or ambition of a project; the scale of population size required to gather the necessary evidence; the duration of the reintroduction trial (i.e. time needed for research versus a sense of urgency or available resource).
