# Supplementary material for: Renewed coexistence: learning from steering group stakeholders on a beaver reintroduction project in England
Source: Eur J Wildl Res. 2021 Dec 3;68(1):1. doi: 10.1007/s10344-021-01555-6 (PMC8640482; doi:10.1007/s10344-021-01555-6)
Supplement: Supplementary file 3 — Supplementary file3 (DOCX 43 KB) [file 10344_2021_1555_MOESM3_ESM.docx]

**SUPPORTING INFORMATION: Research Information and Survey Questions**

**Article**: Renewed Coexistence: Learning from Steering Group Stakeholders on a Beaver Reintroduction Project in England

**Journal**: European Journal of Wildlife Research

**Authors**: Roger E Auster (University of Exeter; [r.e.auster@exeter.ac.uk](mailto:r.e.auster@exeter.ac.uk)), Prof. Stewart Barr (University of Exeter), Prof. Richard Brazier (University of Exeter)


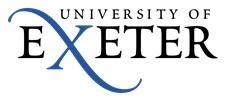


**RESEARCH INVITATION: REVIEW OF THE RIVER OTTER BEAVER TRIAL (ROBT)**

As was proposed to the ROBT Steering Group on 13th February 2020, the University of Exeter would like to invite you to participate in a review of your experiences of involvement in the River Otter Beaver Trial processes. The aim of this research is to record your experiences and views of the ROBT process, and to identify key factors that may help to inform future decisions and processes regarding present and other potential (re)introductions of beavers and other species.

This invitation is to be shared with all members of the Steering Group and, as the lead groups for key document outputs of the ROBT, with members of the Beaver Management Strategy Framework Working Group and Science & Evidence Forum.

**Please Note: We recognise that, due to the Covid-19 outbreak, you may be facing other difficulties at this time. As such, we would like to remind you that undertaking this survey is a voluntary process. We will keep the survey open for an extended period until the 10^th^ August.**

This online questionnaire will ask you to comment in a range of areas including: how you became involved and your role in the Trial; your views of what the Trial has done best; your views on how the Trial may have been improved; what lessons you believe there to be for possible future (re)introductions of beavers or other species.

The outputs of this research will first be reported upon within a findings report for the ROBT, then followed by an academic paper subjected to the peer review process, and within a PhD thesis.

Your contribution towards this work is voluntary and you are not obliged to answer any or all questions should you wish to leave them blank. That which you do contribute is greatly appreciated. Thank you for your participation in this research.

Please note, the questionnaire will be open for completion until **12pm on Monday 10th August 2020**.                                                                                                                        

If you would like to view the questions prior to participation, please click here: insert hyperlink to question list.


**Prior to participation, please read the following ethical and data protection statements.**

**Participation Requirements**
· Participation will involve undertaking this online questionnaire which is estimated to take approximately 30 minutes.
· Taking part in this research is entirely voluntary, and if you choose to withdraw at any time then you may do so by informing the researcher. If you withdraw, all identifiable information that you provided will be removed. You may leave any or all of the questions blank should you wish not to provide an answer.
· The original proposal included the option of additional opt-in interviews following this process if deemed appropriate. The option to opt-in is provided at the end of the questionnaire. Whether interviews are required will be determined following the questionnaire process. In light of the current coronavirus outbreak, any interviews that do take place will be conducted by phone or through a computer software of your choosing (e.g., Skype, Microsoft Teams, Zoom etc.) in order to avoid face-to-face contact.

**Personal Information**
· Your personal details (including email address) will be held confidentially, and therefore won't be shared. Contact details will only be held in order to contact you in relation to this research. Any personal information will be treated as confidential and not be used in a way that would allow for the identification of your individual participation in the research outputs.
· Information on the organisations involved in the ROBT is in the public domain. As such, these organisations may be listed within the research outputs. However, your personal details will not be shared.
· In order to avoid reputational risk to yourself or to your organisation, your comments will not be directly attributed towards your respective organisation unless otherwise stated by you in your responses. If a comment provided by you is directly attributed to an organisation, the researcher will contact you prior to its inclusion in the research outputs in order to confirm you are happy for its usage.

**Outputs and Data Use**
· The outputs of this research will be reported upon within a findings report for the ROBT, an academic paper subjected to the peer review process, and within a PhD thesis. The findings report will be first presented to research participants in order to provide opportunity for comment, prior to the document being published. The following academic paper will be subjected to the scientific peer review process.
· Data will be stored securely and anonymously at the University of Exeter and then shared in a public repository in order to make it available to other researchers in line with current data sharing practices. The shared data will **not** include any personally identifiable information. If you have directly attributed any answers to your organisation, these comments will be redacted unless permission has been granted by yourself for them to be included.

**Funding**
· This study is funded by the University of Exeter, Devon & Cornwall Wildlife Trusts and Plymouth City Council.

**Researcher Contact Details**
· Should you have any questions or queries relating to this research, please feel able to contact the researcher, Roger Auster, at any time on rea213@exeter.ac.uk

Please click here to indicate that you have read and agree to the ethical and data protection statements.

Checkbox. This is required to be checked to proceed in the software.

## **Section 1: Your Details**

These first questions are for the researcher to be able to contact you, should there need to be any follow-up on your comments. **These personal details will not be shared nor presented in any research output.**

Please state your name

Comment box

Please provide a contact email address

Comment box

Did you participate in the Trial as an individual or as a representative of an organisation? In the case of the latter, please state which one.

As stated in the ethical information, your answers will not be directly attributed to your organisation in any research output unless stated by and agreed with yourself.

Comment box

## **Section 2: Your involvement in the Trial**

The next three questions will ask you about which group(s) you sat on. For each one that you indicate, you will then be asked to indicate your role upon that group from a check-list.

**1.** Were you a member of the ROBT Steering Group?

Yes – if selected, show check-list

No – if selected, skip to question 2

Check-list. Please use the checklist to indicate your role upon the Steering Group. You may select as many answers as appropriate.

Chair or Convenor

Contributor – on the ground monitoring or management

Contributor – scientific research

Advisor (to provide advice or ideas, or to be consulted with)

Informer (to inform of wider policy or developments)

Informee (to be kept informed of developments)

Reporter (lead for group output reports)

Funder (contributing funder for the ROBT)

Other – Please specify - Text field

**2.** Were you a member of the ROBT Beaver Management Strategy Framework Working Group (BMSF)?

Yes – if selected, show check-list

No – if selected, skip to next question 3

Check-list. Please use the checklist to indicate your role upon the BMSF. You may select as many answers as appropriate.

Chair or Convenor

Contributor – on the ground monitoring or management

Contributor – scientific research

Advisor (to provide advice or ideas, or to be consulted with)

Informer (to inform of wider policy or developments)

Informee (to be kept informed of developments)

Reporter (lead for group output reports)

Funder (contributing funder for the ROBT)

Other – Please specify - Text field

**3.** Were you a member of the ROBT Science and Evidence (S&E) Forum?

Yes – if selected, show check-list

No – if selected, skip to next question 4

Check-list. Please use the checklist to indicate your role upon the S&E Forum. You may select as many answers as appropriate.

Chair or Convenor

Contributor – on the ground monitoring or management

Contributor – scientific research

Advisor (to provide advice or ideas, or to be consulted with)

Informer (to inform of wider policy or developments)

Informee (to be kept informed of developments)

Reporter (lead for group output reports)

Funder (contributing funder for the ROBT)

Other – Please specify - Text field

**4.** Please state whether you/your organisation holds a particular stance on beaver reintroduction, and whether this has in any way changed over the course of the Trial?

Comment box

**5.** What were the main motivations for you/your organisation to participate in the Trial?

Comment box

**6.** Please describe any potential risks or challenges you believe there to have been of your/your organisation’s participation in the Trial.

Comment box

## **Section 3: Trial Review**

This section will question your views on the Trial itself, and what you feel the Trial may or may not have achieved.

**7.** Do you consider the Trial structure to have been appropriate for conducting the Trial? Please give reasons for your answer, including any changes you would suggest if appropriate.

‘Trial structure’ in this question refers to the existence and relationships between the Licensing Group, Steering Group, Management Strategy Framework Working Group, Science & Evidence Forum, Fisheries Forum and Community Forum. It also refers to the relationship between these groups and the on-the-ground monitoring by Devon Wildlife Trust.

Comment box

**8.** Do you consider the composition of organisations on the group(s) upon which you sat (SG/BMSF/S&E) to have been appropriate? Please give reasons for your answer, including any changes you would suggest, if appropriate.

Please specify which group(s) you are referring to within your response(s).

Comment box

**9.** Please detail here if you believe there to have been any key successes of the Trial.

Comment box

**10.** Please detail here if you believe there to have been any key failures of the Trial.

Comment box

**11.** Overall and on balance, have you found your experience of participating in the Trial to have been of value for you/your organisation?

Yes

No

Please use this space to give reason for your answer to question 11.

Comment box

## **Section 4: Lessons for the Future**

**12.** From your experience of this Trial, what do you believe are the key lessons learned for considering potential future (re)introductions of beavers or other species?

Comment box

**13.** Following your experience of this Trial, please tell us whether or not you believe potential future (re)introductions of beavers or other species should be subject to a similar Trial process. Please give reasons for your answer.

Comment box

**14.** Following your experience from this Trial, would you/your organisation consider participating in potential future trial (re)introductions of beavers or other species? Please give reasons for your answer, including what would incentivise your participation.

Comment box

## **Section 5: Final Additional Comments**

Please use this space to provide any additional comments you may have.

Comment box

Finally, if deemed appropriate, would you be willing to discuss your answers with the researcher in a follow-up interview?

Due to the current coronavirus outbreak, any follow-up interviews will take place through a digital medium to avoid any risk to you or the researcher of viral transmission.

Yes

No


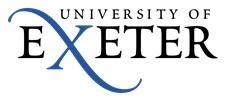


Thank you very much for taking part in this research. We are very grateful for your participation.

As a reminder, the outputs of this research will be reported upon within a findings report for the ROBT, an academic paper subjected to the peer review process, and within a PhD thesis. The findings report will be first presented to research participants in order to provide opportunity for comment in due course, prior to the document being published.

Thank you.

Yours gratefully,

Roger Auster
rea213@exeter.ac.uk
