# Supplementary material for: Renewed coexistence: learning from steering group stakeholders on a beaver reintroduction project in England
Source: Eur J Wildl Res. 2021 Dec 3;68(1):1. doi: 10.1007/s10344-021-01555-6 (PMC8640482; doi:10.1007/s10344-021-01555-6)

# SUPPORTING INFORMATION: Research Proposal Slide

**Article**: Renewed Coexistence: Learning from Steering Group Stakeholders on a Beaver Reintroduction Project in England

**Journal**: European Journal of Wildlife Research

**Authors**: Roger E Auster (University of Exeter; [r.e.auster@exeter.ac.uk](mailto:r.e.auster@exeter.ac.uk)), Prof. Stewart Barr (University of Exeter), Prof. Richard Brazier (University of Exeter)

**NB.** The following was a slide presented to the ROBT Steering Group on 13^th^ February 2020 when making the initial research proposal.


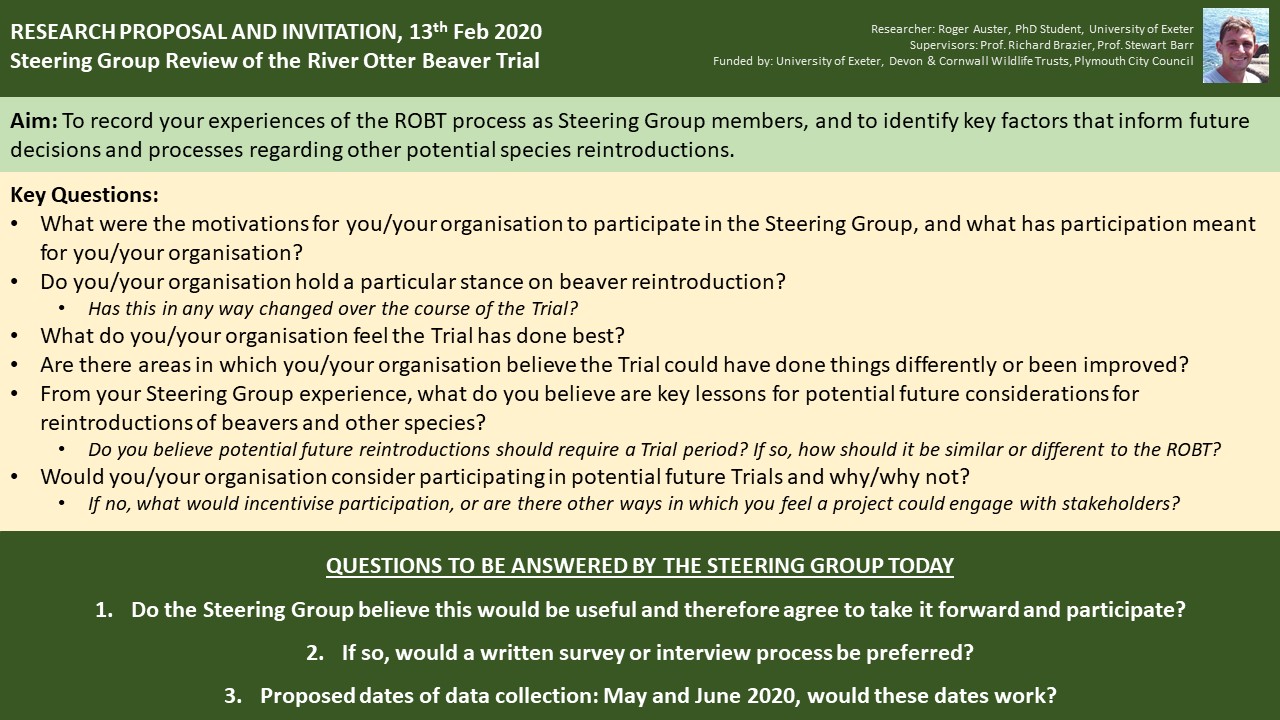

Supplement: Supplementary file 4 — Supplementary file4 (DOCX 264 KB) [file 10344_2021_1555_MOESM4_ESM.docx]
